# Implications of Preoperative Depression for Lumbar Spine Surgery Outcomes: A Systematic Review and Meta-Analysis

**DOI:** 10.1001/jamanetworkopen.2023.48565

**Published:** 2024-01-26

**Authors:** Saad Javeed, Braeden Benedict, Salim Yakdan, Samia Saleem, Justin K. Zhang, Kathleen Botterbush, Madelyn R. Frumkin, Angela Hardi, Brian Neuman, Michael P. Kelly, Michael P. Steinmetz, Jay F. Piccirillo, Burel R. Goodin, Thomas L. Rodebaugh, Wilson Z. Ray, Jacob K. Greenberg

**Affiliations:** 1 Department of Neurological Surgery, Washington University, St Louis, Missouri; 2 Department of Psychology and Brain Sciences, Washington University, St Louis, Missouri; 3 Department of Musculoskeletal Research, Washington University, St Louis, Missouri; 4 Department of Orthopedic Surgery, Washington University, St Louis, Missouri; 5 Department of Anesthesiology, Washington University, St Louis, Missouri; 6 Becker Medical Library, Washington University, St Louis, Missouri; 7 Department of Otolaryngology, Washington University, St Louis, Missouri; 8 Department of Neurological Surgery, Cleveland Clinic, Cleveland, Ohio; 9 Department of Orthopedic Surgery, Rady Children’s Hospital, University of California, San Diego, San Diego

## Abstract

**Importance:**

Comorbid depression is common among patients with degenerative lumbar spine disease. Although a well-researched topic, the evidence of the role of depression in spine surgery outcomes remains inconclusive.

**Objective:**

To investigate the association between preoperative depression and patient-reported outcome measures (PROMs) after lumbar spine surgery.

**Data Sources:**

A systematic search of PubMed, Cochrane Database of Systematic Reviews, Embase, Scopus, PsychInfo, Web of Science, and ClinicalTrials.gov was performed from database inception to September 14, 2023.

**Study Selection:**

Included studies involved adults undergoing lumbar spine surgery and compared PROMs in patients with vs those without depression. Studies evaluating the correlation between preoperative depression and disease severity were also included.

**Data Extraction and Synthesis:**

All data were independently extracted by 2 authors and independently verified by a third author. Study quality was assessed using Newcastle-Ottawa Scale. Random-effects meta-analysis was used to synthesize data, and *I*^2^ was used to assess heterogeneity. Metaregression was performed to identify factors explaining the heterogeneity.

**Main Outcomes and Measures:**

The primary outcome was the standardized mean difference (SMD) of change from preoperative baseline to postoperative follow-up in PROMs of disability, pain, and physical function for patients with vs without depression. Secondary outcomes were preoperative and postoperative differences in absolute disease severity for these 2 patient populations.

**Results:**

Of the 8459 articles identified, 44 were included in the analysis. These studies involved 21 452 patients with a mean (SD) age of 57 (8) years and included 11 747 females (55%). Among these studies, the median (range) follow-up duration was 12 (6-120) months. The pooled estimates of disability, pain, and physical function showed that patients with depression experienced a greater magnitude of improvement compared with patients without depression, but this difference was not significant (SMD, 0.04 [95% CI, −0.02 to 0.10]; *I*^2^ = 75%; *P* = .21). Nonetheless, patients with depression presented with worse preoperative disease severity in disability, pain, and physical function (SMD, −0.52 [95% CI, −0.62 to −0.41]; *I*^2^ = 89%; *P* < .001), which remained worse postoperatively (SMD, −0.52 [95% CI, −0.75 to −0.28]; *I*^2^ = 98%; *P* < .001). There was no significant correlation between depression severity and the primary outcome. A multivariable metaregression analysis suggested that age, sex (male to female ratio), percentage of comorbidities, and follow-up attrition were significant sources of variance.

**Conclusions and Relevance:**

Results of this systematic review and meta-analysis suggested that, although patients with depression had worse disease severity both before and after surgery compared with patients without depression, they had significant potential for recovery in disability, pain, and physical function. Further investigations are needed to examine the association between spine-related disability and depression as well as the role of perioperative mental health treatments.

## Key Points

**Question:**

Is there an association between preoperative depression and the outcomes of lumbar spine surgery?

**Findings:**

In this systematic review and meta-analysis of 44 studies, patients with depression presented with worse spine disease severity, which remained worse after lumbar spine surgery compared with those without depression. However, both groups of patients had a similar magnitude of postoperative improvement in disability, pain, and physical function.

**Meaning:**

Findings of this study suggest that, although patients with depression often had worse disease severity than patients without depression, they typically experience meaningful improvement in spine-related disability after lumbar spine surgery; further investigations are needed to examine the association between spine-related disability and depression as well as the role of perioperative mental health treatments.

## Teaser

This systematic review and meta-analysis describes the association between preoperative depression and postoperative patient-reported disability, pain, and physical function after spine surgery among adults enrolled in 44 studies.

## Introduction

Degenerative lumbar spine disease is one of the most common disabilities associated with substantial loss in quality of life.^1,2^ High-level of evidence suggests that lumbar spine surgery can result in meaningful improvement in symptoms and quality of life in appropriately selected patients.^3,4,5^

A large number of studies have shown that a subset of patients who undergo spine surgery present with comorbid psychiatric disorders such as depression and/or anxiety.^6,7,8^ It is well established that comorbid depression affects the severity of low back pain symptoms and treatment outcomes.^9,10^ Consequently, the association between depression and back pain has been a major focus of personalized treatment studies of the National Institutes of Health Back Pain Consortium.^11,12,13^

Despite this large body of evidence in the broader back pain literature, there have been conflicting results regarding the role of comorbid depression in lumbar surgery outcomes.^7,14,15,16,17,18^ Therefore, establishing the role of depression in lumbar spine surgery outcomes can help inform surgical guidance and adjunctive treatment recommendations.^19^ To address this evidence gap, we performed a systematic review and meta-analysis to investigate the association between preoperative depression and patient-reported outcome measures (PROMs) after lumbar spine surgery. We also examined the methods used to measure preoperative depression to inform the development of uniform standards for evaluating this comorbidity.

## Methods

### Literature Search

An electronic search was performed in PubMed, Cochrane Database of Systematic Reviews, Embase, Scopus, PsychInfo, Web of Science, and ClinicalTrials.gov from database inception to September 14, 2023, using search terms related to mental health or depression and lumbar spine surgery. The search strategy was designed with the aid of a medical research librarian and is described in eTable 1 in Supplement 1. Two of us (S.J., J.K.Z.) independently reviewed the titles and abstracts from identified studies to evaluate their eligibility for inclusion. Conflicts were reconciled by a third author (J.K.G.). We followed the Preferred Reporting Items for Systematic Reviews and Meta-analyses (PRISMA) reporting guideline.^20^

### Eligibility Criteria

We included studies that performed a comparative analysis of PROMs in patients with vs without preoperative depression and/or evaluated the correlation of preoperative depression, defined on a quantitative scale, with preoperative and/or postoperative spinal disease severity. Only studies that involved adults who underwent surgery for degenerative lumbar spine disease were included. It was necessary that depression was defined on a preoperative level. Use of both a validated depression scale and a reported history of diagnosed depression was accepted, given that both approaches have been used in the spine surgery literature. We required that outcomes were measured with PROMs validated in patients undergoing lumbar spine surgery and that PROMs were used both before and at least 6 months after surgery.

We excluded studies that did not measure depression preoperatively, given that postoperative assessment could be confounded by recall bias. Studies on surgeries for cervical spine, tumor, trauma, and/or infection were excluded. Research based on administrative data sets, case reports, literature reviews, and articles that were not published in English were also excluded.

### Data Extraction

Using predesigned forms, 2 of us (B.B., S.Y.) independently extracted both study-level and patient-level data. The first author (S.J.) verified all variables and calculated standardized mean differences (SMDs) to measure the effect size of differences in means between patients with and without depression. Any conflicts were resolved by the senior author (J.K.G.). The interrater reliability between 2 reviewers was quantified using the Cohen κ statistic.^21^ The methods used to classify depression and the PROMs used to measure outcomes are documented in eTables 2 and 3 in Supplement 1.

### Outcome Measures

The primary outcome was the SMD of change in disease severity from the preoperative baseline to the latest postoperative follow-up for patients with vs without depression. Secondary outcomes were the differences in absolute disease severity both before and after surgery in those with vs without depression classification as well as the linear correlation between depression severity and severity in spine-related disability both preoperatively and postoperatively. Mean, SD, and number of patients in each group were required to calculate the SMD. In studies reporting median and IQR or 95% CI, variables were transformed to mean and SD according to the Cochrane guidelines.^22^

Given the heterogeneity of the PROMs used to evaluate patients with lumbar surgery, we consolidated closely related measures assessing the same underlying constructs (eg, numeric pain severity scores). The details and rationale for these decisions are provided in eTable 4 in Supplement 1. The PROMs included in the meta-analyses had established measurement properties and had been validated in patients with spine surgery.^23,24,25,26,27^ Before the meta-analysis, we transformed all PROMs so that all measures were scored in the same direction, meaning that a lower score represented lower disease severity. For studies reporting the association between quantitative depression measures and PROMs, both Pearson correlation coefficients and β coefficients from regression analysis were used and integrated using the established methods described in the eMethods in Supplement 1.^28^ All correlation coefficients were converted to the same direction and transformed into Fisher *z* coefficients for meta-analysis.^29^

### Study Quality

The quality of each study was rated using the Newcastle-Ottawa Scale.^30^ Along with the study quality, other study-level factors, such as design, journal impact factor, level of evidence, and quality of analyses, were also captured.

### Statistical Analysis

Effect sizes were calculated from the outcome means and SDs and were converted to SMDs to evaluate between-group differences.^31^ Since multiple methods were used to define depression and evaluate lumbar disease severity, there was substantial heterogeneity across studies. Therefore, random-effects meta-analysis was used.^32^ The heterogeneity was quantified using *I*^2^ statistics and visualized with forest plots. Subgroup meta-analyses were performed according to types of disease severity measures, evaluating the same primary and secondary outcome measures.

To evaluate the potential role of heterogeneity (*I*^2^) in the association of depression with outcomes, we performed sensitivity analyses using metaregression. We extracted several variables that could account for heterogeneity in outcomes across studies. These variables included patient-level, study-level, and intervention-level factors that may alter the interpretation of our results. The association of each variable with the outcome was evaluated using univariable metaregression. Any variable with *R*^2^ greater than 0 in univariable analysis was included in the multivariable metaregression analysis, and age and sex (male to female ratio) were tested for clinical importance. To evaluate the potential publication bias secondary to small-study effects, we used funnel plots and the Egger test of asymmetry.

All analyses were conducted in R, version 4.2.1 (R Project for Statistical Computing). Meta-analysis and metaregression were performed with the metafor package, version 4.1.4, in R. The threshold of significance was set at 2-tailed α < .05.

## Results

### Study Characteristics

The initial search yielded 8459 articles (eFigure 1 in Supplement 1). After removing duplicates, 3813 articles were screened, leaving 207 for full-text review. Among these studies, 44 met the eligibility criteria; each study is described in eTable 5 in Supplement 1.^6,7,8,14,15,16,17,18,33,34,35,36,37,38,39,40,41,42,43,44,45,46,47,48,49,50,51,52,53,54,55,56,57,58,59,60,61,62,63,64,65,66,67,68^ Overall, 21 452 patients were included, of whom there were 11 747 females (55%) and 9705 males (45%) with a mean (SD) age of 57 (8) years. Two studies involved a mix of lumbar and cervical populations and were excluded from all quantitative meta-analyses.

Aggregated study characteristics are given in the Table. Of the 44 studies included, 15 (34%) had a prospective design and 29 (66%) had a retrospective design. Most studies (36 [82%]) were conducted in a single center, while 8 (18%) were in multicenters. Additionally, 27 studies (61%) performed a comparative analysis of surgical outcomes between patients with and without depression, 7 (16%) evaluated the correlation between quantitative depression and spine severity measures, and 10 (23%) performed both analyses (Table). The median (range) duration of follow-up was 12 (6-120) months. Among comparative studies (n = 37), 37% of patients had preoperative depression.

**Table.** Study Characteristics

Variable^a^	No. (%) (n = 44)
Patient characteristics	
Sample size	21 452
Age, mean (SD), y	57 (8)
Sex	
Males	9705 (45)
Females	11 747 (55)
Comorbidities, mean (SD), %	31 (24)
Follow-up duration, median (range), mo	12 (6-120)
Spine surgery characteristics	
Surgery levels	
Single	12 (27)
Multiple	25 (57)
Not reported	7 (16)
Surgery type	
Fusion^b^	25 (57)
Fusion plus decompression only^c^	18 (41)
Not reported	1 (2)
Surgical approach	
Posterior	31 (71)
Anteroposterior	11 (25)
Not reported	2 (4)
Study characteristics	
Design	
Prospective	15 (34)
Retrospective	29 (66)
Centers	
Single	36 (82)
Multiple	8 (18)
Type of analysis	
Comparative	27 (61)
Correlation^d^	7 (16)
Both	10 (23)
Mental health screening method	
Quantitative	37 (84)
Medical record diagnosis	7 (16)
Quality of methods	
Reported surgery details	37 (84)
Reported handling of missing data	7 (16)
Multivariable analysis	26 (59)
Adjusted covariates	
Age	24 (54)
Sex	22 (50)
Comorbidities	20 (45)
Baseline symptom severity	16 (36)
Socioeconomic status	11 (25)
Propensity matching	2 (4)
Sensitivity analysis	9 (20)
Publication characteristics	
Impact factor, median (IQR)	3 (2-3)
Publication year	
2005-2010	3 (7)
2011-2015	8 (18)
2016-2023	34 (75)

^a^ All characteristics represent study-level data. For example, mean age represents average of the mean ages in studies; SD represents variation in means across studies.

^b^ Studies including patient population undergoing spine fusion procedures.

^c^ Studies including patient population undergoing spine fusion and decompression only procedures.

^d^ Correlation between quantitative depression and spine severity measures.

### Reporting Quality

Details of the surgical interventions (eg, number of levels treated) were reported in 37 studies (84%), methods for how missing data were handled were reported in 7 studies (16%), and multivariable analyses were conducted in 26 studies (59%). However, nearly half of the studies did not account for several covariates known to be factors in outcomes of lumbar spine surgery, such as age (24 [54%]), comorbidities (20 [45%]), and baseline symptom severity (16 [36%]) (Table).^69^

Overall, the study quality was moderate, with a median (IQR) Newcastle Ottawa Scale score of 7 (5-9) (eTable 6 in Supplement 1). There was substantial heterogeneity in the methods used to diagnose depression. Among comparative studies (n = 37), 30 (81%) used quantitative scales and 7 (19%) used the depression diagnosis in patient’s medical record. The most common quantitative method used was the Short Form Mental Component Summary (SF-MCS) scores, which was used in 11 studies (29%) (eTable 2 in Supplement 1). Among studies that used common depression scales, 20 (54%) used variable cutoffs to stratify depression diagnosis (eTable 2 in Supplement 1). In terms of outcomes, most studies reported primary outcomes related to disability, pain, or physical function. The most common PROM used was the Oswestry Disability Index (35 studies [79%]), followed by the Visual Analog Scale for back (22 studies [50%]) and leg (20 studies [45%]) pain scores. The full list of PROMs with frequencies is given in eTable 3 in Supplement 1. Overall, there was a substantial interrater reliability between the 2 independent reviewers (eTable 7 in Supplement 1).

### Depression-Related Comorbidity in Spine Disease

**Figure 1.**
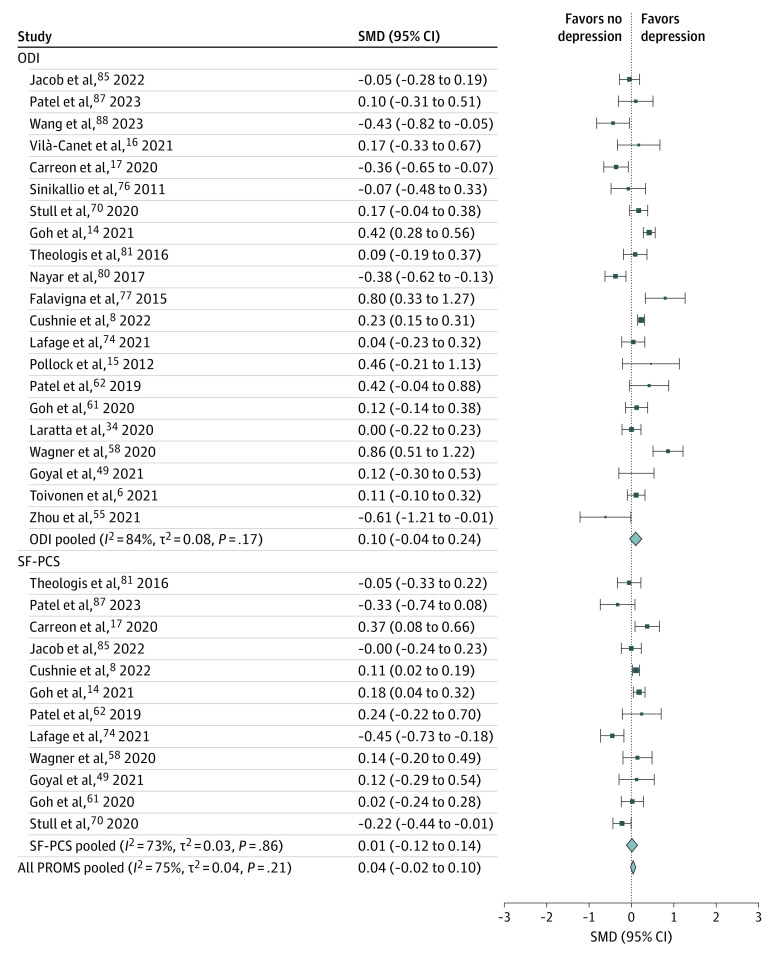
Meta-Analysis of Primary Outcomes and Improvement in Disability and Physical Function in Patients Without vs With Preoperative Depression Size of squares indicates the weight of each study, error bars indicate 95% CIs of standardized mean difference (SMD), and diamonds indicate pooled estimates of patient-reported outcome measure (PROM) subgroups and all PROMs with 95% CIs. The *I*^2^, *t*^2^, and *P* values were calculated using a random-effects model. All PROMS pooled represents the comparison of all outcomes combined (Oswestry Disability Index [ODI], Short Form Physical Component Score [SF-PCS], Visual Analog Scale scores for back and leg pain).

**Figure 2.**
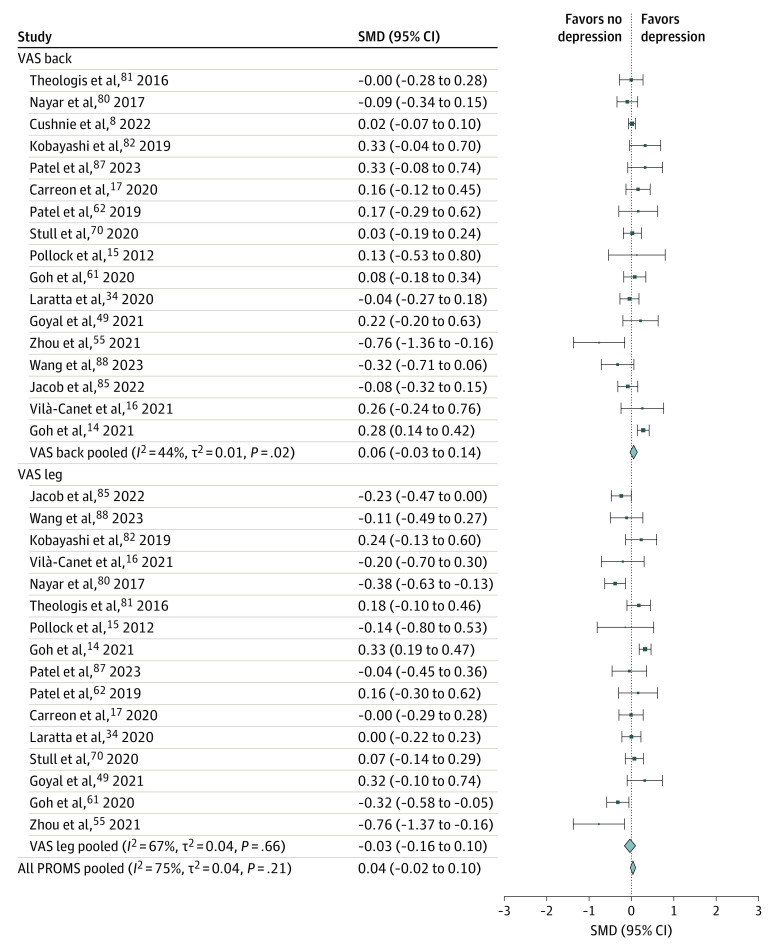
Meta-Analysis of Primary Outcomes and Improvement in Back and Leg Pain in Patients Without vs With Preoperative Depression Size of squares indicates the weight of each study, error bars indicate 95% CIs of standardized mean difference (SMD), and diamonds indicate pooled estimates of patient-reported outcome measure (PROM) subgroups and all PROMs with 95% CIs. The *I*^2^, *t*^2^, and *P* values were calculated using a random-effects model. All PROMS pooled represents the comparison of all outcomes combined (Oswestry Disability Index [ODI], Short Form Physical Component Score [SF-PCS], Visual Analog Scale [VAS] scores for back and leg pain).

Overall, there was significant heterogeneity in the estimates for primary and secondary outcomes (*I*^2^ ranged from 44% to 99%). Regarding the primary outcome, there was no significant difference in the SMD of change in disability, pain, and physical function from preoperative baseline to postoperative follow-up in patients with vs without depression (SMD, 0.04 [95% CI, −0.02 to 0.10]; *I*^2^ = 75%; *P* = .21). Patients with depression had a larger magnitude of improvement from baseline to latest follow-up compared with patients without depression, but this difference was not significant (Figure 1, Figure 2). Regarding secondary outcomes, despite experiencing greater improvement after surgery, patients with depression had worse preoperative disability, pain, and physical function compared with patients with depression at baseline (SMD, −0.52 [95% CI, −0.62 to −0.41]; *I*^2^ = 89%; *P* < .001), which remained worse postoperatively (SMD, −0.52 [95% CI, −0.75 to −0.28]; *I*^2^ = 98%; *P* < .001) (eFigure 2 in Supplement 1).

### Sensitivity Analyses

Next, we examined the heterogeneity of the results. The *I*^2^ was high (range, 44%-84%), which reflected high between-study variability for the primary outcome. To ascertain whether study-level, patient-level, or intervention-level factors could explain this variance, we performed a metaregression analysis.

Among all variables, 5 explained the significant heterogeneity across studies in univariable metaregression (eTable 8 in Supplement 1): percentage of comorbidities, first year of patient enrollment, follow-up attrition, depression stratification method, and level of surgery. Along with these variables, age and sex were tested a priori and ultimately were included in the multivariable analysis because they were associated with substantial improvement in the overall variance explained (*R*^2^ = 75% [included in multivariable model] vs 38% [not included in multivariable model]). In the final multivariable model, older age and larger male to female ratio were associated with larger SMDs, whereas higher percentage of comorbidities, later study year, and higher follow-up attrition rates were associated with smaller SMDs. The *I*^2^ (ie, between-study variability due to unaccounted-for heterogeneity) was 48% (*P* < .001) (eTable 8 in Supplement 1).

Among the studies comparing change in disease severity in patients with vs without depression, 7 (19%) used depression diagnosis from medical records as a screening method. However, depression diagnosis may not accurately reflect depression severity or capture current depressive symptoms. To further investigate, we conducted a sensitivity analysis evaluating the use of depression diagnosis vs quantitative scale as a screening method. In both analyses, there was no significant difference in postoperative change in disease severity among patients with vs without depression (eFigure 3 in Supplement 1).

Eleven studies (29%) used SF-MCS as a preoperative depression stratification method (eTable 3 in Supplement 1). Although SF-MCS has shown good performance in measuring depression burden in general and in patients with spine surgery in particular, this PROM is not depression specific.^70^ Therefore, we performed a sensitivity analysis to evaluate the implications of using SF-MCS as a depression stratification method. Regardless of whether SF-MCS or a depression-specific scale was used to classify depression, there was no significant difference in the change in PROMs after surgery among patients with vs without depression (eFigure 4 in Supplement 1).

### Correlation Between Depression and Outcome Measures

**Figure 3.**
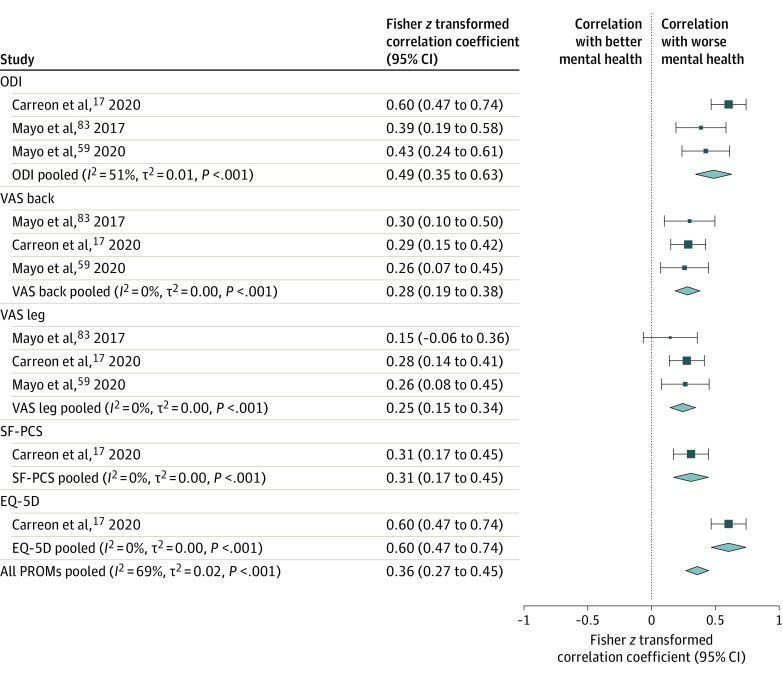
Meta-Analysis of Correlations Between Preoperative Quantitative Depression Measures and Preoperative and Postoperative Patient-Reported Outcome Measures (PROMs) Increased values indicate worsening PROMs and directly correlate with worse preoperative depression. Size of squares indicates the weight of each study, error bars indicate 95% CIs of Fisher *z* correlation coefficients, and diamonds indicate pooled estimates of PROM subgroups and all PROMs with 95% CIs. The *I*^2^, *t*^2^, and *P* values were calculated using a random-effects model. EQ-5D indicates EuroQol 5-Dimension; ODI, Oswestry Disability Index; SF-PCS, Short Form Physical Component Score; VAS, Visual Analog Scale.

**Figure 4.**
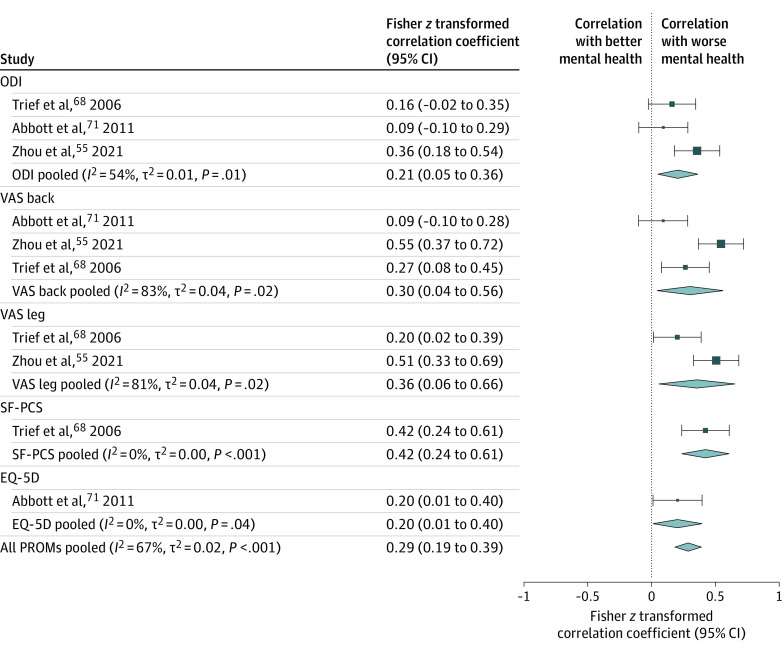
Meta-Analysis of Correlations Between Preoperative Quantitative Depression Measures and Postoperative Patient-Reported Outcome Measures (PROMs) Increased values indicate worsening of PROMs and directly correlate with worse preoperative depression. Size of squares indicates the weight of each study, error bars indicate 95% CIs of Fisher *z* correlation coefficients, and diamonds indicate pooled estimates of PROM subgroups and all PROMs with 95% CIs. The *I^2^*, *t*^2^, and *P* values were calculated using a random-effects model. EQ-5D indicates EuroQol 5-Dimension; ODI, Oswestry Disability Index; SF-PCS, Short Form Physical Component Score; VAS, Visual Analog Scale.

The correlation of preoperative depression measured on a quantitative scale with baseline and postoperative disease severity is shown in Figure 3 and Figure 4. Pooled estimates revealed a correlation between depression and disability, pain, physical function, and quality-of-life outcomes at baseline (Fisher *z* coefficient = 0.36 [95% CI, 0.27-0.45]; *I*^2^ = 69%; *P* < .001) and at postoperative follow-up (Fisher *z* coefficient = 0.29 [95% CI, 0.19-0.39]; *I*^2^ = 67%; *P* < .001). However, there was no correlation between preoperative depression severity and change in PROMs from baseline to postoperative follow-up (Fisher *z* coefficient = −0.05 [95% CI, −0.15 to 0.05]; *I*^2^ = 93%; *P* = .36) (eFigure 5 in Supplement 1).

### Publication Bias

Visual inspection of the funnel plot for the primary outcome showed moderate asymmetry (eFigure 6 in Supplement 1). There was no evidence of small-study effect or publication bias (Egger test *z* = −0.84; *P* = .40). However, there were several studies within the region of significance (ie, *P* < .05).^71^ This finding suggests that the observed asymmetry was due to factors, such as inconsistent study quality and/or depression measures, leading to heterogeneous findings.

## Discussion

This systematic review and meta-analysis synthesized the evidence regarding the association of depression with lumbar spine surgery outcomes. We found that patients with preoperative depression experienced a similar magnitude of improvement in spine-related disability, pain, and physical function compared with patients without depression. Despite this finding, patients with depression still had worse absolute disease severity in both preoperative and postoperative settings. This finding is consistent with reports that patients with depression experience worse disease severity before surgery and may have greater room for recovery after surgery.^34,72,73,74^ At the same time, the association between depression severity and preoperative and postoperative spine disease severity supports the role of impaired mental health in lumbar spine care. Furthermore, this study highlighted the inconsistent methods used to diagnose preoperative depression, emphasizing the need for standardized methods to assess this comorbidity.

Depression is prevalent in the US,^75^ commonly occurring alongside chronic musculoskeletal pain,^76,77,78^ particularly in patients with chronic low back pain.^9,79^ Depression is associated with increased pain, disability, longer symptom duration, and poor treatment response in patients.^11,80^ Although the precise association between depression and pain-related disability remains unknown, several possible mechanisms have been proposed.^76,81,82,83^ This association may be bidirectional,^84^ such that comorbid depression is associated with catastrophizing followed by amplification of pain and pain-related disability.^82,85^ Alternatively, prior depression itself can precede the worsening of spine-related pain and disability.^9,78^ Although the deleterious association of depression with pain-related disability has been well studied, the extent to which depression affects surgical outcomes remains poorly defined. This knowledge gap impairs effective patient counseling and perioperative efforts to maximize surgical outcomes.

The findings of this study may provide timely estimates regarding the implications of comorbid depression for lumbar surgery outcomes. Although patients with depression presented with worse disease severity, they had a comparable or possibly even larger magnitude of improvement after lumbar spine surgery. This finding aligns with results published in primary care and musculoskeletal pain literature showing that the treatment of chronic painful conditions is also associated with substantial improvement in depression.^86^ While these findings support the role of spine surgery in patients with comorbid depression, patients with depression typically exhibit more severe preoperative symptoms, which allows for a greater potential for postoperative improvement.^34,72,73,74^ Therefore, this study not only supports the role of appropriately indicated lumbar spine surgery in patients with depression but also emphasizes mental health treatment in this population.

In addition to evaluating differences in disease severity and postoperative outcomes between patients with and without a depression classification, this study quantified the linear correlation between depression severity and spine-related disability severity. Similar to outcomes comparing patients on the basis of dichotomous depression classifications, worse spine-related disability was associated with worse depression severity. However, there was no association between preoperative depression severity and postoperative change in spine-related disability, similar to the primary analysis using dichotomous depression classifications. One explanation for this finding is that depression severity is not associated with postoperative change in spine-related disability. Alternatively, there may be a nonlinear association between depression severity and postoperative recovery, which this analysis was unable to explain.

There was significant heterogeneity in the estimates (*I*^2^ range, 44%-84%). Several factors accounted for this heterogeneity, which aligns with previous studies. For example, a larger percentage of common comorbidities was associated with smaller SMDs in improvement among patients with vs without depression. This finding highlights that factors, such as frailty and surgical invasiveness, may confound the role of depression in surgical outcome.^69^ Additionally, older age was associated with larger SMDs, potentially reflecting the fact that depression symptoms may be more severe in patients 70 years or older.^87^ The diminished role of depression reported in more recent studies^6,15,17,33,38,39,43,44,62,65^ suggests that better-quality depression treatment may help mitigate its association with surgical outcomes. These findings have implications for the spine surgery literature and related investigations in other surgical fields.

This study also highlighted the lack of standardized tools for depression screening, which has been a major focus of the Depression Screening Data initiative.^88^ The initiative recommends the use of Patient Health Questionnaire-9 (PHQ-9) for depression screening in primary care settings.^89^ However, only 4 of 37 studies (10%) used PHQ-9,^7,8,36,42^ emphasizing the need to standardize practices in spine surgery research.

### Strengths and Limitations

This study has some strengths, including the thorough search strategy, robust evidence synthesis, and rigorous exploration of confounding factors. Nevertheless, the study has several limitations. First, there was substantial heterogeneity across studies. Although several variables accounted for a large proportion of variance in the estimates, this inconsistency may have limited the conclusions drawn. Similarly, heterogeneous depression screening methods may have introduced additional variability in the analysis. Second, due to lack of reported data, it was not feasible to study the outcome of co-occurring mental health conditions, such as anxiety or kinesiophobia, which may affect the role of depression in surgical outcomes (eTable 9 in Supplement 1). Third, information regarding active or prior depression treatment was not available, which may have confounded the analyses.

## Conclusions

In this systematic review and meta-analysis, we found that patients with depression presented with worse spine disease severity, which remained worse after lumbar spine surgery, compared with patients without depression. However, these patients may experience a similar magnitude of postoperative improvement, reflecting their significant potential for improvement in spine disability, pain, and physical function. Further investigations are needed to examine the causal association between spine-related disability and depression as well as the role of perioperative mental health treatments.

## Notes

Supplement 1. **eFigure 1.** PRISMA Flowchart for Study Selection **eFigure 2.** Meta-Analysis of Secondary Outcomes in Patients With vs. Without Depression **eFigure 3.** Sensitivity Analysis of Studies Using Depression Diagnosis as Screening Method **eFigure 4.** Sensitivity Analysis of Studies Using SF MCS as Depression Stratification Method **eFigure 5.** Correlation of Depression Measures With Primary Outcomes **eFigure 6.** Funnel Plot of All Studies Including Primary Outcomes **eMethods.** Use and Transformation of Correlations Data for Meta-Analysis **eTable 1.** Systematic Search Strategy **eTable 2.** Depression Screening Methods in Studies Performing Comparative Analysis **eTable 3.** Patient Reported Outcome Measures in Included Studies **eTable 4.** Conversion of PROMs for Comparison and Inclusion in Meta-Analysis **eTable 5.** Characteristics of Included Studies **eTable 6.** Study Quality and Risk of Bias Using the Newcastle-Ottawa Scale **eTable 7.** Inter-Rater Reliability of Screened and Extracted Data **eTable 8.** Meta-Regressions to Identify the Variables Explaining *I^2^* in Improvement in PROMs in Patients With No-Depression vs Depression **eTable 9.** Non-Depression Mental Health Conditions Assessed in Included Studies
eReferences

Supplement 2.
Data Sharing Statement
