# Supplementary material for: Implications of Preoperative Depression for Lumbar Spine Surgery Outcomes: A Systematic Review and Meta-Analysis
Source: JAMA Netw Open. 2024 Jan 26;7(1):e2348565. doi: 10.1001/jamanetworkopen.2023.48565 (PMC10818221; doi:10.1001/jamanetworkopen.2023.48565)
Supplement: Supplement 1. — eFigure 1. PRISMA Flowchart for Study Selection eFigure 2. Meta-Analysis of Secondary Outcomes in Patients With vs. Without Depression eFigure 3. Sensitivity Analysis of Studies Using Depression Diagnosis as Screening Method eFigure 4. Sensitivity Analysis of Studies Using SF MCS as Depression Stratification Method eFigure 5. Correlation of Depression Measures With Primary Outcomes eFigure 6. Funnel Plot of All Studies Including Primary Outcomes eMethods. Use and Transformation of Correlations Data for Meta-Analysis eTable 1. Systematic Search Strategy eTable 2. Depression Screening Methods in Studies Performing Comparative Analysis eTable 3. Patient Reported Outcome Measures in Included Studies eTable 4. Conversion of PROMs for Comparison and Inclusion in Meta-Analysis eTable 5. Characteristics of Included Studies eTable 6. Study Quality and Risk of Bias Using the Newcastle-Ottawa Scale eTable 7. Inter-Rater Reliability of Screened and Extracted Data eTable 8. Meta-Regressions to Identify the Variables Explaining I2 in Improvement in PROMs in Patients With No-Depression vs Depression eTable 9. Non-Depression Mental Health Conditions Assessed in Included Studies eReferences [file jamanetwopen-e2348565-s001.pdf]

Supplemental Online Content

Javeed S, Benedict B, Yakdan S, et al. Implications of preoperative depression for lumbar spine surgery outcomes: a systematic review and meta-analysis. *JAMA Netw Open*. 2024;7(1):e2348565. doi:10.1001/jamanetworkopen.2023.48565

eFigure 1. PRISMA Flowchart for Study Selection

eFigure 2. Meta-Analysis of Secondary Outcomes in Patients With vs. Without Depression

eFigure 3. Sensitivity Analysis of Studies Using Depression Diagnosis as Screening Method

eFigure 4. Sensitivity Analysis of Studies Using SF MCS as Depression Stratification Method

eFigure 5. Correlation of Depression Measures With Primary Outcomes

eFigure 6. Funnel Plot of All Studies Including Primary Outcomes

eMethods. Use and Transformation of Correlations Data for Meta-Analysis

eTable 1. Systematic Search Strategy

eTable 2. Depression Screening Methods in Studies Performing Comparative Analysis

eTable 3. Patient Reported Outcome Measures in Included Studies

eTable 4. Conversion of PROMs for Comparison and Inclusion in Meta-Analysis

eTable 5. Characteristics of Included Studies

eTable 6. Study Quality and Risk of Bias Using the Newcastle-Ottawa Scale

eTable 7. Inter-Rater Reliability of Screened and Extracted Data

eTable 8. Meta-Regressions to Identify the Variables Explaining I^2 in Improvement in PROMs in Patients With No-Depression vs. Depression

eTable 9. Non-Depression Mental Health Conditions Assessed in Included Studies

eReferences

This supplemental material has been provided by the authors to give readers additional information about their work.

eFigure 1: PRISMA Flowchart for Study Selection

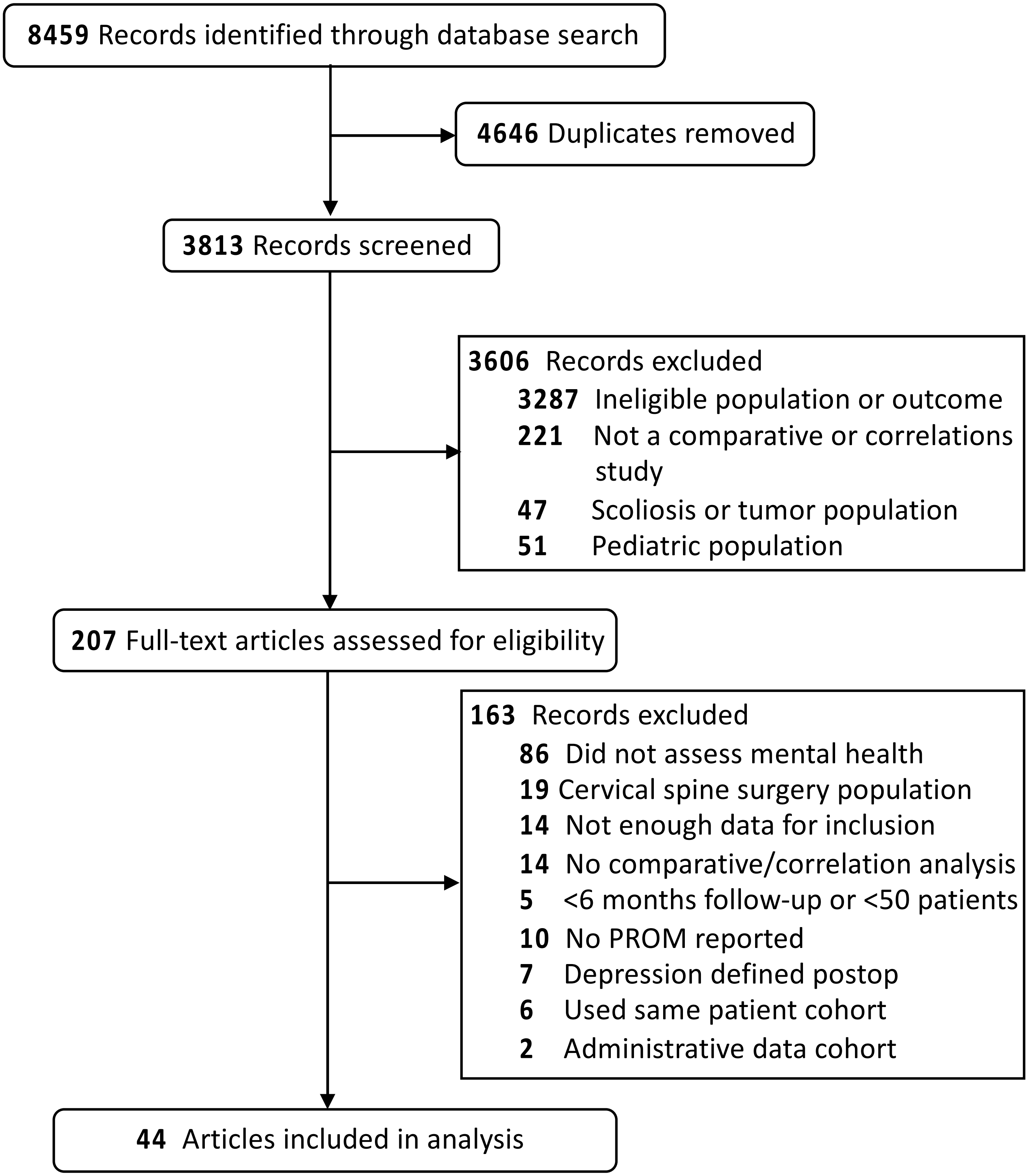

© 2024 Javeed S et al. *JAMA Network Open*

eFigure 2: Meta-analysis of Secondary Outcomes in Patients with vs. without Depression

Secondary Outcome, Absolute Disease Severity Before and After Surgery

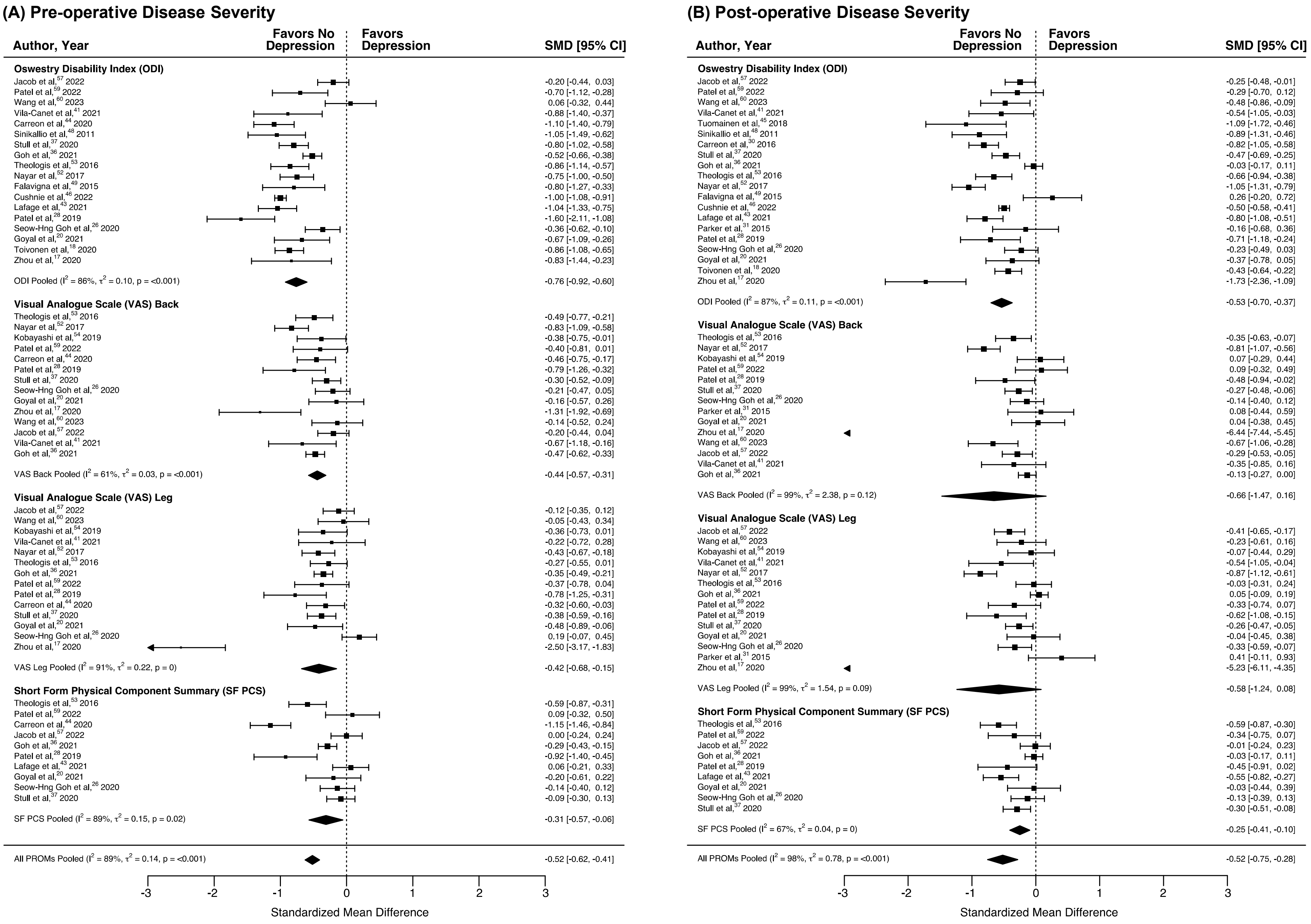

© 2024 Javeed S et al. *JAMA Network Open*

eFigure 3: Sensitivity Analysis of Studies using Depression Diagnosis as Screening Method

eFigure 3A

Primary Outcome, change in disease severity in studies using quantitative scale for depression stratification (excluding studies using depression diagnosis)

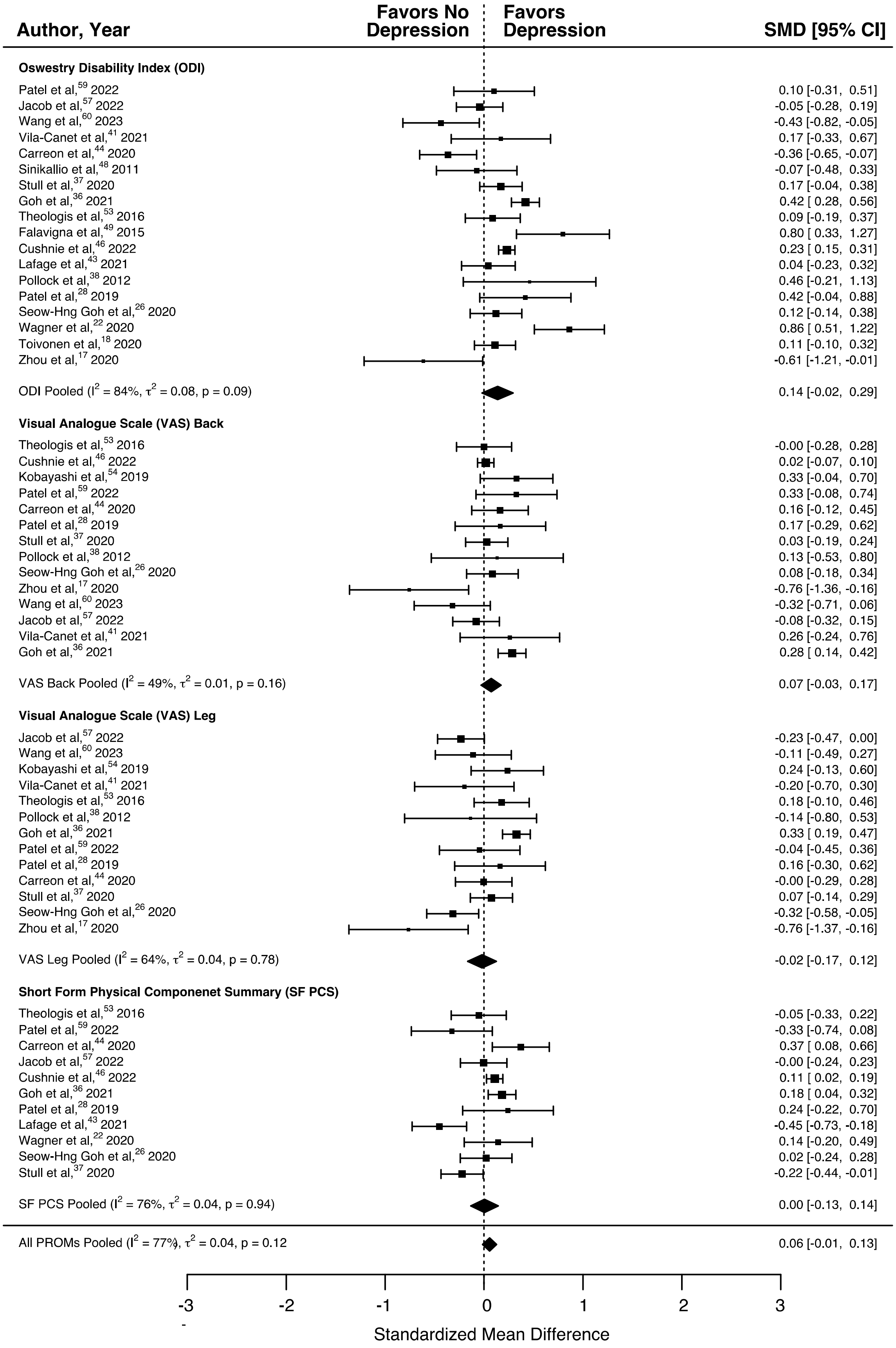

© 2024 Javeed S et al. *JAMA Network Open*

eFigure 3B

Primary Outcome, change in disease severity in studies using medical record diagnosis for depression stratification

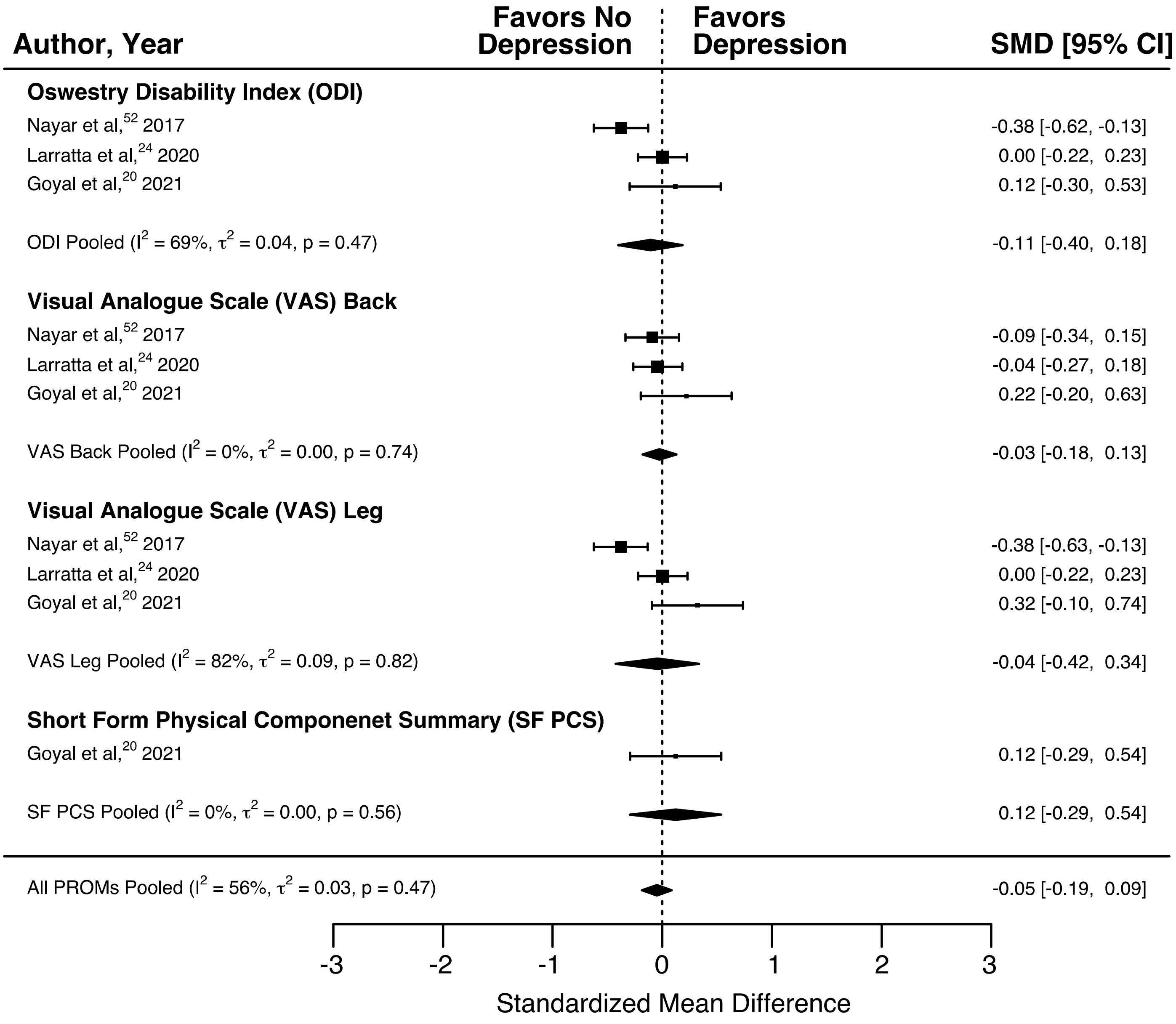

© 2024 Javeed S et al. *JAMA Network Open*

eFigure 4: Sensitivity Analysis of Studies using SF MCS as Depression Stratification Method

eFigure 4A

Primary Outcome, change in disease severity in studies using depression-specific scale for depression stratification (excluding studies using SF MCS)

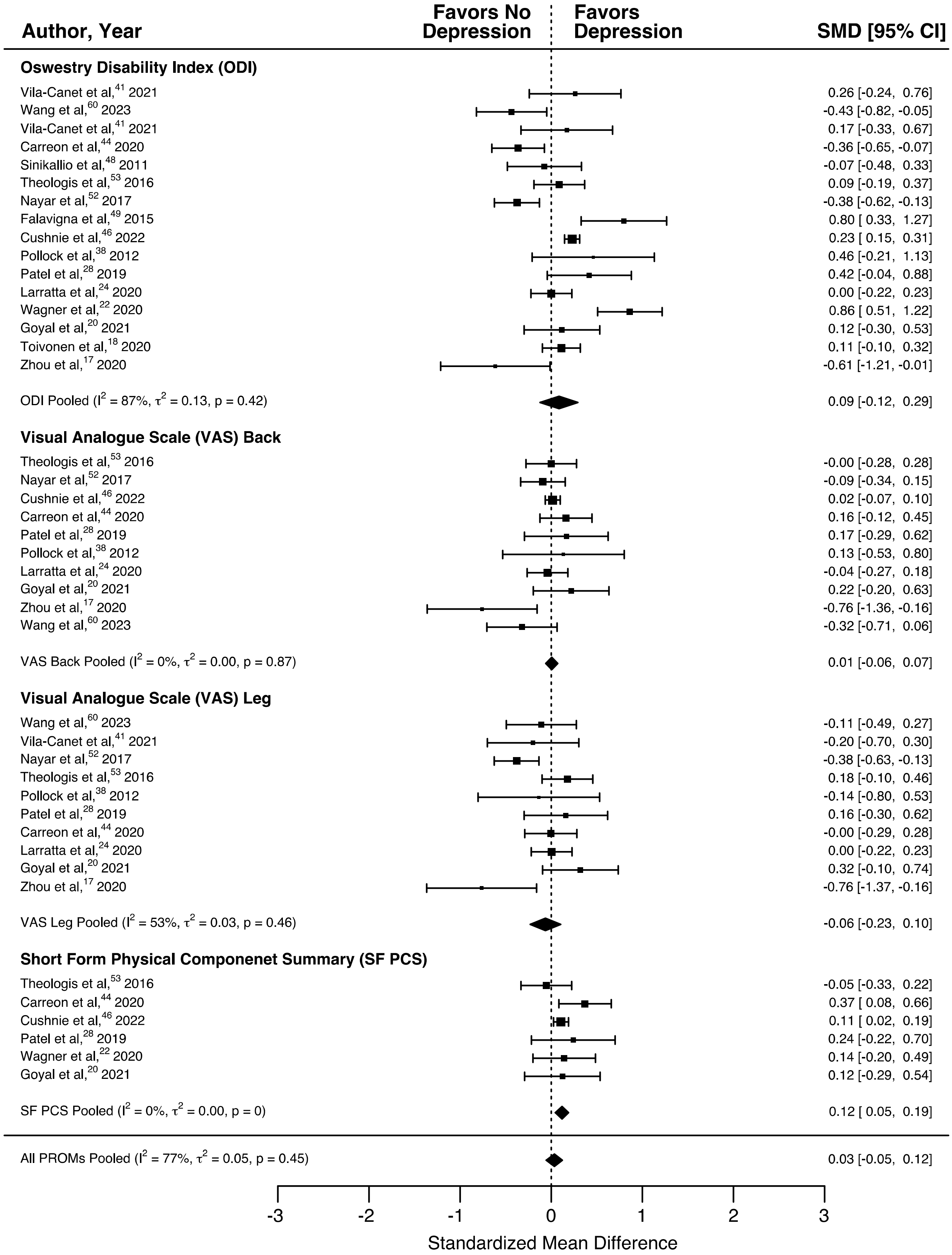

© 2024 Javeed S et al. *JAMA Network Open*

eFigure 4B

Primary Outcome, change in disease severity in studies using SF MCS for depression stratification

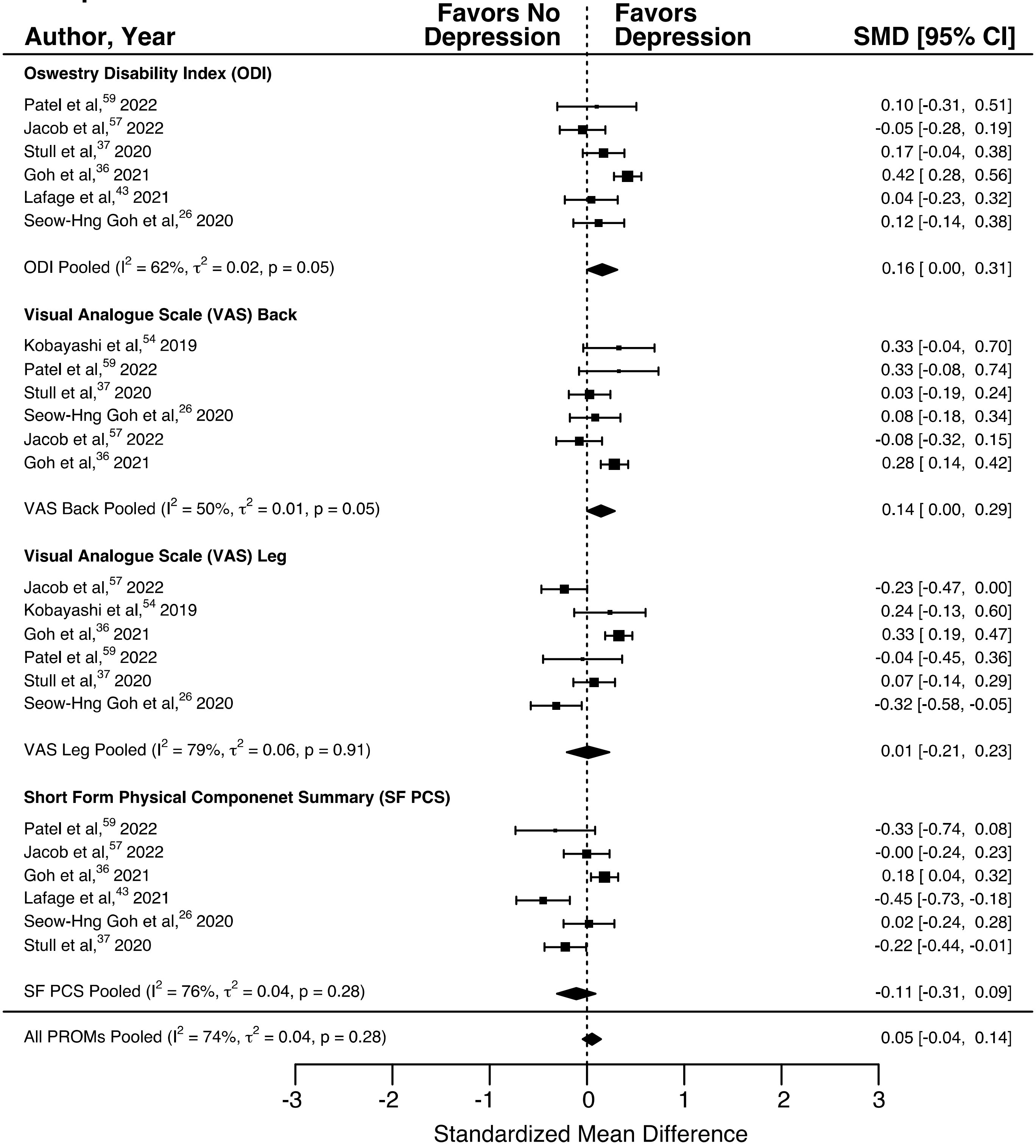

© 2024 Javeed S et al. *JAMA Network Open*

eFigure 5: Correlation of Depression Measures with Primary Outcomes

Correlation of depression scales with improvement in PROMs

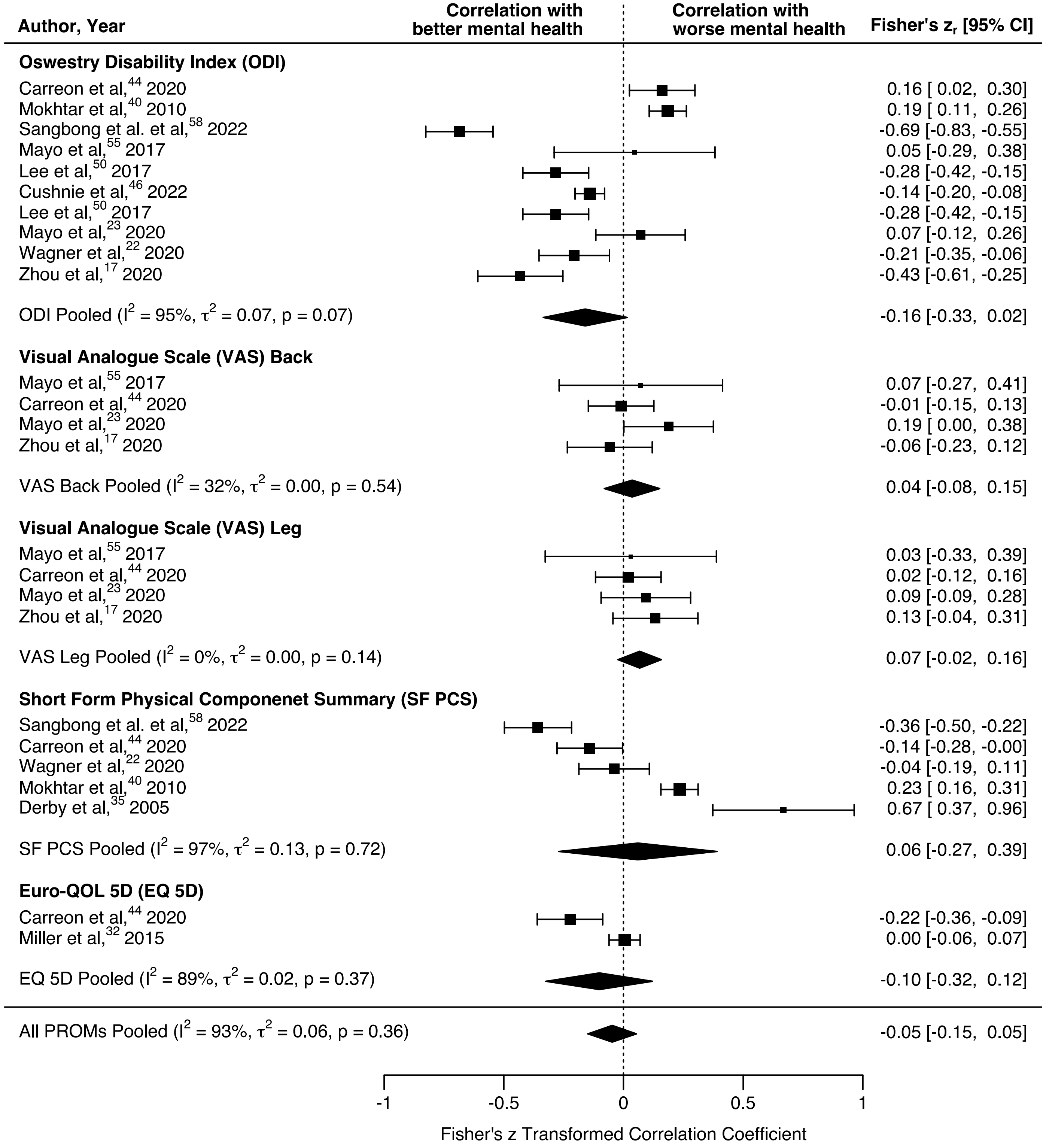

© 2024 Javeed S et al. *JAMA Network Open*

eFigure 6: Funnel Plot of All Studies Including Primary Outcomes

Funnel plot, Egger test, z= -0.84, p=0.40. Shaded regions indicate levels of significance. The white region indicate p values greater than 0.10, the dark gray-shaded region indicate p values between 0.10 and 0.05, the medium gray-shaded region indicate p values between 0.05 and 0.01, and the region outside of the funnel indicate p values below 0.01.[1] The presence of several studies within the regions of significance (i.e., p<0.05) suggests that the observed asymmetry is likely result of factors other than publication bias such as variable study quality and/or methods.[1]

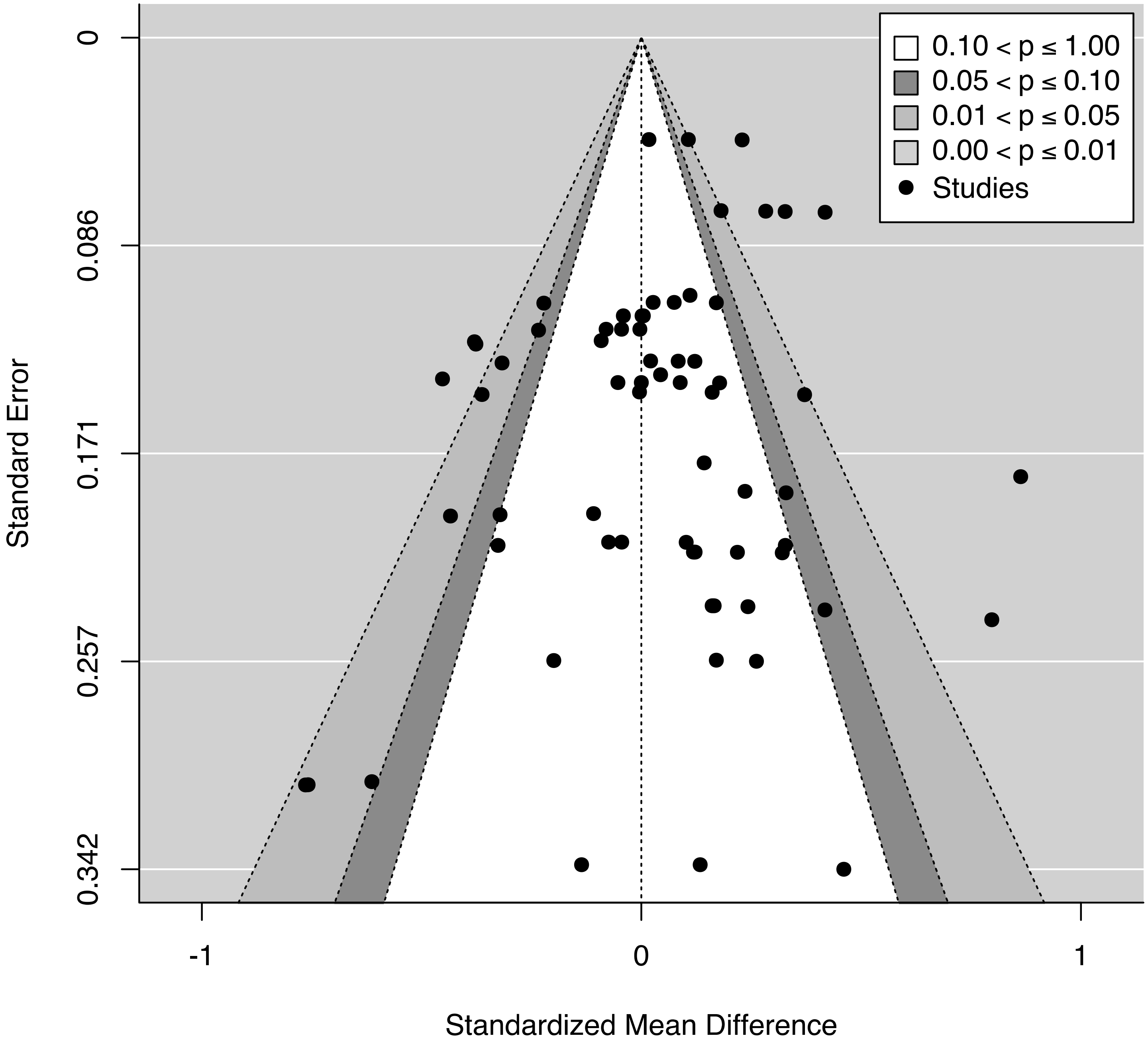

© 2024 Javeed S et al. *JAMA Network Open*

eMethods: Use and Transformation of Correlations Data for Meta-Analysis

We calculated the correlation coefficients based on the criteria described by Peterson and Brown.[2] The following criteria was used for correlations data:

Correlation coefficients (r) eligible:

- Pearson's correlation.
- β coefficient from univariable linear regression.
- β coefficient from multivariable linear regression.
- The depression must be measured on a scale.

Correlation coefficients (r) ineligible:

- Spearman's correlation.
- β coefficients from logistic regression.

While some studies reported standardized β regression coefficients, others reported unstandardized regression coefficients, which vary in magnitude depending on the predictor and outcome variables.[2] Unstandardized coefficients, b, were transformed to standardized coefficients, β, using the standard deviations (either directly reported or estimated from the data)[3,4] for the predictor and outcome variables, s_x and s_y, respectively[5]:

$$\beta = \frac{s_x}{s_y} b$$

This conversion results in a unitless β coefficient which can be used to estimate the correlation coefficient. The following equation was used to transform β coefficients to correlation coefficients[2]:

$$r = \beta + 0.05\lambda$$

In which λ is an indicator variable that equals 1 when is β is positive and 0 when it is negative.[2]

Pearson's correlation coefficients were used as is. After calculating all transformed correlation coefficients and converting them in same direction (i.e., positive correlations correspond to correlation of worse depression with worse disease severity), we used *escalc()* function in *metafor (R)* to transform correlation coefficients to Fisher *z* coefficients for meta-analysis.[6]

© 2024 Javeed S et al. *JAMA Network Open*

eTable 1: Systematic Search Strategy			
**#**	**Database**	**Search terms**	**Results**
1	Embase.com	('spine fusion'/exp OR ‘lumbar fusion*’:ti,ab OR ‘lumbar spine fusion*’:ti,ab OR ‘lumbar interbody fusion*’:ti,ab OR ‘spinal fusion*’:ti,ab OR ‘spine fusion*’:ti,ab OR ‘vertebral fusion*’:ti,ab OR spondylodesis:ti,ab OR spondylodeses:ti,ab OR spondylosyndesis:ti,ab OR spondylosyndeses:ti,ab) AND (('mental health'/exp OR 'depression'/exp OR 'anxiety'/de OR 'anxiety disorder'/exp) AND ('preoperative period'/exp OR 'postoperative period'/de) OR (‘Mental Component Score’:ti,ab OR ‘Mental Component Summary’:ti,ab OR ‘health-care related quality of life’:ti,ab OR ‘health care related quality of life’:ti,ab OR ‘HRQOL’:ti,ab OR ‘visual analog scale’:ti,ab) OR ((‘mental health’ OR ‘mental condition’ OR ‘mental status’ OR ‘mental state’ OR ‘mental status’ OR depression OR depressive OR anxiety OR ‘acute stress disorder*’ OR ‘distress syndrome*’ OR ‘posttraumatic stress disorder*’) NEAR/4 (preoperative OR prior OR pre-existing OR preexisting OR ‘before surger*’ OR ‘before operation*’ OR postoperative OR post-operative OR “post operation” OR “post surger*” OR “post surgical”)):ti,ab) AND [english]/lim	2,534
2	Ovid-Medline All	(Spinal Fusion/ OR "lumbar fusion*".ti,ab. OR "lumbar spine fusion*".ti,ab. OR "lumbar interbody fusion*".ti,ab. OR "spinal fusion*".ti,ab. OR "spine fusion*".ti,ab. OR "vertebral fusion*".ti,ab. OR spondylodesis.ti,ab. OR spondylodeses.ti,ab. OR spondylosyndesis.ti,ab. OR spondylosyndeses.ti,ab.) AND ((Mental Health/ OR Depression/ OR exp Depressive Disorder/ OR Anxiety/ OR exp Anxiety Disorders/) AND (Preoperative Period/ OR Postoperative Period/) OR ("Mental Component Score".ti,ab. OR "Mental Component Summary".ti,ab. OR "health-care related quality of life".ti,ab. OR "health care related quality of life".ti,ab. OR "HRQOL".ti,ab. OR "visual analog scale".ti,ab.) OR (("mental health" OR "mental condition" OR "mental status" OR "mental state" OR "mental status" OR depression OR depressive OR anxiety OR "acute stress disorder*" OR "distress syndrome*" OR "posttraumatic stress disorder*") adj4 (preoperative OR prior OR pre-existing OR preexisting OR "before surger*" OR "before operation*" OR postoperative OR post-operative OR "post operation" OR "post surger*" OR "post surgical")).ti,ab.)	2,161
3	APA PsycInfo	(“lumbar fusion*” OR “lumbar spine fusion*” OR “lumbar interbody fusion*” OR “spinal fusion*” OR “spine fusion*” OR “vertebral fusion*” OR spondylodesis OR spondylodeses OR spondylosyndesis OR spondylosyndeses) AND (("Mental Component Score" OR "Mental Component Summary" OR "health-care related quality of life" OR "health care related quality of life" OR "HRQOL" OR "visual analog scale") OR (("mental health" OR "mental condition" OR "mental status" OR "mental state" OR "mental status" OR depression OR depressive OR anxiety OR "acute stress disorder*" OR "distress syndrome*" OR "posttraumatic stress disorder*") N4 (preoperative OR prior OR pre-existing OR preexisting OR "before surger*" OR "before operation*" OR postoperative OR post-operative OR "post operation" OR "post surger*" OR "post surgical")))	38
4	Scopus	TITLE-ABS ("lumbar fusion*" OR "lumbar spine fusion*" OR "lumbar interbody fusion*" OR "spinal fusion*" OR "spine fusion*" OR "vertebral fusion*" OR spondylodesis OR spondylodeses OR spondylosyndesis OR spondylosyndeses) AND (TITLE-ABS("Mental Component Score" OR "Mental Component Summary" OR "health-care related quality of life" OR "health care related quality of life" OR "HRQOL" OR "visual analog scale") OR TITLE-ABS (("mental health" OR "mental condition" OR "mental status" OR "mental state" OR "mental status" OR depression OR depressive OR anxiety OR "acute stress disorder*" OR "distress syndrome*" OR "posttraumatic stress disorder*") W/4 (preoperative OR prior OR pre-existing OR preexisting OR "before surger*" OR "before operation*" OR postoperative OR post-operative OR "post operation" OR "post surger*" OR "post surgical"))) AND (LIMIT-TO (LANGUAGE , "English")) AND (EXCLUDE (DOCTYPE , "no") OR EXCLUDE (DOCTYPE , "ch") OR EXCLUDE (DOCTYPE , "le"))	1,661
4	ClinicalTrials.gov	(EXPAND[Concept] "mental health" OR EXPAND[Concept] "mental status" OR EXPAND[Concept] "mental state" OR depression OR depressive OR anxiety OR EXPAND[Concept] "stress disorder") AND AREA[ConditionSearch] (lumbar fusion OR spinal fusion OR spine fusion OR spondylodesis)	62

(continued) eTable 1: Systematic Search Strategy			
#	Database	Search terms	Results
6	Web of Science Core Collection	Indexes=SCI-EXPANDED, SSCI, A&HCI, ESCI Timespan=All years<br>1 TOPIC: (("lumbar fusion*" OR "lumbar spine fusion*" OR "lumbar interbody fusion*" OR "spinal fusion*" OR "spine fusion*" OR "vertebral fusion*" OR spondylodesis OR spondylodeses OR spondylosyndesis OR spondylosyndeses))<br>2 TOPIC: (("Mental Component Score" OR "Mental Component Summary" OR "health-care related quality of life" OR "health care related quality of life" OR "HRQOL" OR "visual analog scale"))<br>3 TOPIC: ((("mental health" OR "mental condition" OR "mental status" OR "mental state" OR "mental status" OR depression OR depressive OR anxiety OR "acute stress disorder*" OR "distress syndrome*" OR "posttraumatic stress disorder*") NEAR/4 (preoperative OR prior OR pre-existing OR preexisting OR "before surger*" OR "before operation*" OR postoperative OR post-operative OR "post operation" OR "post surger*" OR "post surgical")))<br>4 3 OR 2<br>5 4 AND 1	1,394
7	Cochrane Library	Cochrane Review and CENTRAL Trials	671
7	1	MeSH descriptor: [Spinal Fusion] explode all trees	974
	2	(“lumbar spine fusion*” OR “lumbar interbody fusion*” OR “spinal fusion*” OR “spine fusion*” OR “vertebral fusion*” OR spondylodesis OR spondylodeses OR spondylosyndesis OR spondylosyndeses):ti,ab,kw	2315
	3	1 OR 2	2315
	4	MeSH descriptor: [Mental Health] explode all trees	1654
	5	MeSH descriptor: [Depression] explode all trees	12932
	6	MeSH descriptor: [Depressive Disorder] explode all trees	12630
	7	MeSH descriptor: [Anxiety] explode all trees	8306
	8	MeSH descriptor: [Anxiety Disorders] explode all trees	7202
	9	4 OR 5 OR 6 OR 7 OR 8	33633
	10	MeSH descriptor: [Preoperative Period] explode all trees	319
	11	MeSH descriptor: [Postoperative Period] explode all trees	6054
	12	10 OR 11	6288
	13	9 AND 12	192
	14	(("Mental Component Score" OR "Mental Component Summary" OR "health-care related quality of life" OR "health care related quality of life" OR "HRQOL" OR "visual analog scale")):ti,ab,kw	53033
	15	((("mental health" OR "mental condition" OR "mental status" OR "mental state" OR "mental status" OR depression OR depressive OR anxiety OR "acute stress disorder*" OR "distress syndrome*" OR "posttraumatic stress disorder*") NEAR/4 (preoperative OR prior OR pre-existing OR preexisting OR "before surger*" OR "before operation*" OR postoperative OR post-operative OR "post operation" OR "post surger*" OR "post surgical"))):ti,ab,kw	2797
	16	13 OR 14 OR 15	55511
	17	3 AND 16	543

© 2024 Javeed S et al. *JAMA Network Open*

eTable 2: Depression Screening Methods in Studies performing Comparative Analysis		
**Measure**	**Description**	**N (%) (N=37)**
SF-MCS[a]	Short form 8, 12, or 36 mental component summary	11 (29)
PHQ	Patient health questionnaire- 2 or 9	4 (10)
BDI[b]	Beck Depression Inventory	4 (10)
ZDS[c]	Zung depression scale	5 (13)
HADS	Hospital anxiety and depression scale	3 (8)
CESDS-ADS-K	Center for Epidemiological Studies Depression Scale (Allgemeine Depression Scale)	1 (2)
DEPS	Depression Scale	1 (2)
PROMIS-Depression	Patient reported information system depression domain	3 (8)
History of Depression	Self-reported history of depression	1 (2)
Diagnosis of Depression	Diagnosis; No depression and depression	6 (16)

Variable cut-offs were used in studies: [a]SF-MCS: <36, <40, <45, and <50; [b]BDI: >=10 and >=15; [c]ZDS: >33 and >50

© 2024 Javeed S et al. *JAMA Network Open*

eTable 3: Patient Reported Outcome Measures in Included Studies			
**PROM**	**Description**	**Domain Assessed**	**N (%) (N=44)**
ODI[7,8]	Oswestry Disability Index	Disability	35 (79)
VAS Back[9,10]	Visual Analogue Scale for Back Pain	Pain	22 (50)
VAS Leg[9,10]	Visual Analogue Scale for Leg Pain	Pain	20 (45)
SF-PCS[11]	Short Form Physical Component Summary	Quality of life	17 (38)
SF-MCS	Short Form Mental Component Summary	Quality of life	13 (29)
EQ-5D	Euro-Qol-5D	Quality of life	9 (20)
VAS	Visual Analogue Scale for general pain	Pain	8 (18)
BDI	Beck depression inventory	Mental health	3 (7)
NASS	North American satisfaction survey	Satisfaction	3 (7)
PROMIS PF	PROMIS physical function	Function	4 (9)
PROMIS PI	PROMIS pain interference	Disability	1 (2)
PROMIS SD	PROMIS sleep disturbance	Sleep	1 (2)
PROMIS SS	PROMIS social satisfaction	Satisfaction	1 (2)
SSM	Spinal stenosis measure	Function	2 (4)
JOA	Japanese orthopedic association	Disability	1 (2)
LBOS	Low back pain outcome score	Disability	1 (2)
PHQ-9	Patient health questionnaire 9	Mental health	1 (2)
SF-36 BP	Short form 36 bodily pain	Pain	1 (2)
SF-36 PF	Short form 36 physical function	Function	1 (2)
SRS	Scoliosis research society	Quality of life	2 (4)
Stucki	Stucki questionnaire	Disability	1 (2)
ZCQ	Zurick claudication questionnaire	Disability	1 (2)

© 2024 Javeed S et al. *JAMA Network Open*

eTable 4: Conversion of PROMs for comparison and inclusion in meta-analysis		
**PROM[a] Combined**	**PROMS**	**Rationale**
**SF-PCS**	VR-12 PCS, SF-12 PCS, and SF-36 PCS	Though VR-12, SF-12, and SF-36 have minor differences, they were combined because:<br><br>1) summary scores have same comparable range (i.e., 1-100).[12,13]<br><br>2) they are highly correlated reflecting similar concept.[14,15]
**SF-MCS**	VR-12 MCS, SF-12 MCS, and SF-36 MCS	
**VAS**	VAS Back and Leg; VAS non-specified, VAS worst, EQ-5D VAS	All studies reporting VAS either on scale of 1-10 or 1-100 and in different direction were transformed on a scale of 1-10, with increasing points indicating worse symptoms.[16]

Abbreviations:
EQ-5D: EuroQOL 5D; PROMs: Patient Reported Outcome Measures; SF-PCS: Short Form Physical Component Summary; SF-MCS: Short Form Mental Component Summary; VAS: Visual Analogue Scale; VR-12: Veterans Rand 12

© 2024 Javeed S et al. *JAMA Network Open*

eTable 5: Characteristics of Included Studies													
**Source**	**Country**	**Study Period**	**Design**	**Data Source**	**Single or Multi Center**	**Total Pt. (N)**	**Pt. Age (mean)**	**Pt. Sex M (%)**	**Follow-up (Mo)**	**Depression Measure**	**Depressed Group (N)**	**Non-Depressed Group (N)**	**Outcome Measures**
Zhou et al,[17] 2020	China	2016-2018	RCS	Chart review	Single	125	50	66	12	BDI	12	113	ODI, VAS leg, VAS back
Toivonen et al,[18] 2020	Finland	2010-2015	PCS	Prospective Database	Multi	392	61	29	60	Depression Scale (DEPS)	137	255	ODI
Parrish et al,[19] 2021	USA	2016-2018	RCS	Chart review	Single	75	50	56	6	PHQ-9	37	22	PHQ-9
Goyal et al,[20] 2021	USA	2013-2017	RCS	Chart review	Single	391	62	54	12	Diagnosis of Depression	24	323	ODI, VAS back, VAS leg, SF-12 PCS, SF-12 MCS
Yoo et al,[21] 2019	USA	2015-2018	RCS	Chart review	Single	172	51	60	12	SF-12 MCS	85	87	PROMIS PF
Wagner et al,[22] 2020	Germany	2013-2017	PCS	Institutional Registry	Single	180	63	39	12	Allgemeine Depressionsskala (ADS-K)	43	137	ODI, VAS, EQ-5D, SF-36 PCS
Mayo et al,[23] 2020	USA	2013-2016	RCS	Prospective Database	Single	113	53	67	6	SF-12 MCS	56	57	ODI, VAS back, VAS leg
Laratta et al,[24] 2020	USA	2014-2016	RCS	Prospective Database (QOD)	Multi	462	60	31	12	Diagnosis of Depression	111	245	ODI, VAS back, VAS leg, EQ-5D, SF-12 MCS
Jenkins et al,[25] 2019	USA	2006-2018	RCS	Chart review	Single	215	54	58	12	PHQ-9	–	–	SF-12 MCS
Goh et al,[26] 2020	Singapore	2004-2013	RCS	Institutional Registry	Single	296	58	32	60	SF-36 MCS	113	113	ODI, VAS back, VAS leg, SF-36 PCS, SF-36 MCS, NASS neurogenic symptoms
Zakaria et al,[27] 2019	USA	2015-2018	RCS	Prospective Database	Multi	8585	61	45	24	PHQ-2	507	1107	ODI, NASS
Patel et al,[28] 2019	USA	2016-2017	RCS	Chart review	Single	94	54	59	6	PHQ-9	25	69	ODI, VAS back, VAS leg, VR-12 PCS, VR-12 MCS
Amaral et al,[29] 2017	Brazil	2011-2016	RCS	Chart review	Single	136	54	49	12	Diagnosis of Depression	51	85	ODI, VAS, EQ-5D
Carreon et al,[30] 2016	USA	2012-2014	RCS	Prospective Database (QOD)	Single	312	59	44	12	Diagnosis of Depression	58	254	ODI, EQ-5D, SF-36 MCS
Parker et al,[31] 2015	USA	–	PCS	Chart review	Single	58	54	29	24	Zung depression scale (ZDS)	30	28	ODI, EQ-5D, VAS back, VAS leg

© 2024 Javeed S et al. *JAMA Network Open*

(continued) **eTable 5: Characteristics of Included Studies**													
**Source**	**Country**	**Study Period**	**Design**	**Data Source**	**Single or Multi Center**	**Total Pts. (N)**	**Pt. Age (mean)**	**Pt. Sex M (%)**	**Follow-up (Mo)**	**Depression Measure**	**Depressed Group (N)**	**Non-depressed Group (N)**	**Outcome Measures**
Miller et al,[32] 2015	USA	2008-2012	RCS	Chart review	Single	919	60	51	6	PHQ-9	–	–	EQ-5D
Adogwa et al,[33] 2014	USA	2009-2011	PCS	Chart review	Single	69	70	36	24	Zung depression scale (ZDS)	–	–	ODI
Trief et al,[34] 2006	USA	–	PCS	Prospective Database (RCT)	Multi	160	44	52	24	SF-36 MCS	–	–	ODI, VAS back, VAS leg, SF-36 PCS
Derby et al,[35] 2005	USA	1994-2000	RCS	Prospective Database	Single	57	43	-	12	SF-36 MCS	17	30	SF-36 PCS
Goh et al,[36] 2021	Singapore	2006-2015	RCS	Prospective Database	Single	799	61	33	24	SF-36 MCS	436	363	ODI, VAS back, VAS leg, SF-12 PCS, SF-36 PCS, SF-36 MCS
Stull et al,[37] 2020	USA	2013-2017	RCS	Chart review	Single	391	62	54	12	SF-12 MCS	123	268	ODI, VAS back, VAS leg, SF-12 PCS
Pollock et al,[38] 2012	UK	2004-2008	PCS	Prospective Database	Single	74	46	34	24	Zung depression scale (ZDS)	10	64	ODI, VAS back, VAS leg
Abbott et al,[39] 2011	Sweden	2005-2007	PCS	Randomize d Clinical Trial	Single	107	51	38	24	SF-36 Mental Health Subscale	–	–	ODI, EQ-5D, VAS back
Mokhtar et al,[40] 2010	Australia	2000-2008	PCS	Prospective Database	Single	638	66	39	12	SF-12 MCS	–	–	ODI, SF-12 PCS, SF-12 MCS, Low Back Outcome Score (LBOS)
Vila-Canet et al,[41] 2021	Spain	2014-2016	PCS	Prospective Database	Single	97	56	56	12	Zung Depression Scale (ZDS)	19	78	ODI, VAS back, VAS leg
Rahman et al,[42] 2020	USA	2014-2019	PCS	Prospective Database	Single	206	60	49	12	PROMIS Depression	50	156	PROMIS PI, PF, SD, SS
Lafage et al,[43] 2021	USA	2009-2016	RCS	Prospective Database	Multi	513	58	21	24	SF-36 MCS	104	104	ODI, SRS-22R, SF-36 PCS, SF-36 MCS
Carreon et al,[44] 2020	Denmark	–	PCS	Prospective Database	Single	208	59	49	12	HADS-d	72	136	ODI, VAS back, VAS leg, EQ-5D, SF-36 PCS, SF-36 MCS

© 2024 Javeed S et al. *JAMA Network Open*

(continued) **eTable 5: Characteristics of Included Studies**													
**Source**	**Country**	**Study Period**	**Design**	**Data Source**	**Single or Multi Center**	**Total Pts. (N)**	**Pt. Age (mean)**	**Pt. Sex M (%)**	**Follow-up (Mo)**	**Depression Measure**	**Depressed Group (N)**	**Non-depressed Group (N)**	**Outcome Measures**
Tuomainen et al,[45] 2018	Finland	2001-2013	PCS	Surgical Enrollment	Single	70	59	39	120	BDI	13	57	ODI, VAS, BDI
Cushnie et al,[46] 2022	Canada	–	PCS	Prospective Database (CSORN)	Multi	2310	60	50	12	PHQ-9	1013	1297	ODI, VAS back, EQ-5D, SF-12 PCS
Held et al,[47] 2022	Switzer-land	–	PCS	Prospective Database	Multi	401	73	50	24	HADS-d	72	329	Spinal Stenosis Measure (SSM) Symptoms and Function
Sinikallio et al,[48] 2011	Finland	2001-2004	PCS	Surgical Enrollment	Single	96	62	41	24	BDI	48	45	ODI, VAS, Stucki, BDI
Falavigna et al,[49] 2015	Brazil	2009-2011	PCS	Surgical Enrollment	Single	91	50	40	12	BDI	26	65	ODI, BDI
Lee et al,[50] 2017	South Korea	2010-2013	RCS	Prospective registry	Single	206	62	33	24	HADS-d	–	–	ODI, VAS
Maratos et al,[51] 2012	UK	2000-2006	PCS	Prospective registry	Single	302	55	56	24	HADS-d	117	185	SF-36 Physical Functioning and Back Pain
Nayar et al,[52] 2017	USA	–	RCS	Chart review	Single	275	54	53	12	Diagnosis of Depression	101	174	ODI, VAS back, VAS leg, EQ-5D
Theologis et al,[53] 2016	USA	–	RCS	Prospective registry	Multi	267	56	16	24	Self-report history of Depression	66	201	ODI, VAS back, VAS leg, SF-36 PCS, SF-36 MCS, Scoliosis Research Society (SRS-22r)
Kobayashi et al,[54] 2019	Japan	2014-2016	RCS	Chart review	Single	122	70	63	24	SF-8 MCS	47	75	VAS back, VAS leg, JOA, Zurich Claudication Questionnaire (ZCQ)
Mayo et al,[55] 2017	USA	2013-2015	RCS	Prospective registry	Single	110	41	68	6	SF-12 MCS	27	28	ODI, VAS back, VAS leg
Holbert et al,[56] 2022	USA	2019-2021	RCS	Chart review	Single	596	61	52	6	Diagnosis of Depression	133	208	PROMIS PF
Jacob et al,[57] 2022	USA	2016-2021	RCS	Chart review	Single	297	45	38	12	SF-12 MCS	111	186	ODI, VAS back, VAS leg, SF-12 PCS, SF-12 MCS
Sangbong et al,[58] 2022	Korea	2010-2019	RCS	Chart review	Single	198	70	29	12	SF-36 MCS	-	-	ODI, SF-36 PCS, Rolland Morris Disability Questionaire (RMDQ)

© 2024 Javeed S et al. *JAMA Network Open*

(continued) **eTable 5: Characteristics of Included Studies**													
**Source**	**Country**	**Study Period**	**Design**	**Data Source**	**Single or Multi Center**	**Total Pts. (N)**	**Pt. Age (mean)**	**Pt. Sex M (%)**	**Follow-up (Mo)**	**Depression Measure**	**Depressed Group (N)**	**Non-depressed Group (N)**	**Outcome Measures**
Patel et al,[59] 2022	USA	NA	RCS	Chart review	Single	93	47	83	24	SF-12 MCS	48	45	ODI, VAS back, VAS leg, SF-12 PCS, SF-12 MCS, PROMIS PF
Wang et al,[60] 2023	China	2018-2020	RCS	Chart review	Single	231	80	43	12	Zung Depression Scale (ZDS)	30	201	ODI, VAS back, VAS leg
Abbreviations: PCS: Prospective cohort study; RCS: Retrospective cohort study													

© 2024 Javeed S et al. *JAMA Network Open*

eTable 6: Study Quality and Risk of Bias using the Newcastle-Ottawa Scale

Study Author, year	Study Design	Selection				Comparability	Outcome			Total Score
		Exposed Cohort Representative?	Selection of Non-Exposed Cohort	Ascertainment of Exposure	Outcome not present at study start?	Based on Design or Analysis	Assessment of Outcome	Timing of Follow-up (≥6 months)	Adequate Follow-up (≥80%)	
Zhou et al,[17] 2020	Retrospective Cohort	-	+	+	+	++	+	+	+	**8**
Toivonen et al,[18] 2020	Prospective Cohort	+	+	+	+	++	+	+	-	**8**
Parrish et al,[19] 2021	Retrospective Cohort	-	+	+	+	-	+	+	-	**5**
Goyal et al,[20] 2021	Retrospective Cohort	-	+	+	+	++	+	+	+	**8**
Yoo et al,[21] 2019	Retrospective Cohort	-	+	+	+	-	+	+	+	**6**
Wagner et al,[22] 2020	Prospective Cohort	+	+	+	+	++	+	+	-	**8**
Mayo et al,[23] 2020	Retrospective Cohort	-	+	+	+	++	+	+	-	**7**
Laratta et al,[24] 2020	Retrospective Cohort	+	+	+	+	-	+	+	-	**6**
Jenkins et al,[25] 2019	Retrospective Cohort	-	+	+	+	-	+	+	-	**5**
Goh et al,[26] 2020	Retrospective Cohort	-	+	+	+	++	+	+	+	**8**
Zakaria et al,[27] 2019	Retrospective Cohort	+	+	+	+	++	+	+	-	**8**
Patel et al,[28] 2019	Retrospective Cohort	-	+	+	+	+	+	-	+	**6**
Amaral et al,[29] 2017	Retrospective Cohort	-	+	+	+	-	+	+	+	**6**
Carreon et al,[30] 2016	Retrospective Cohort	+	+	+	+	++	+	+	+	**9**
Parker et al,[31] 2015	Prospective Cohort	+	+	+	+	-	+	+	-	**6**
Miller et al,[32] 2015	Retrospective Cohort	-	+	+	+	++	+	+	-	**7**
Adogwa et al,[33] 2014	Prospective Cohort	+	+	+	+	++	+	+	+	**9**
Trief et al,[34] 2006	Prospective Cohort	+	+	+	+	++	+	+	-	**8**
Derby et al,[35] 2005	Retrospective Cohort	-	+	+	+	-	+	+	+	**6**
Goh et al,[36] 2021	Retrospective Cohort	-	+	+	+	++	+	+	+	**8**
Stull et al,[37] 2020	Retrospective Cohort	-	+	+	+	++	+	+	+	**8**

© 2024 Javeed S et al. *JAMA Network Open*

(continued) eTable 6: Study Quality and Risk of Bias using the Newcastle-Ottawa Scale										
**Study Author, year**	**Study Design**	**Selection**				**Comparability**	**Outcome**			**Total Score**
		**Exposed Cohort Representative?**	**Selection of Non-Exposed Cohort**	**Ascertainment of Exposure**	**Outcome not present at study start?**	**Based on Design or Analysis**	**Assessment of Outcome**	**Timing of Follow-up (≥6 months)**	**Adequate Follow-up (≥80%)**	
Pollock et al,[38] 2012	Prospective Cohort	+	+	+	+	-	+	+	+	**7**
Abbott et al,[39] 2011	Prospective Cohort	+	+	+	+	++	+	+	+	**9**
Mokhtar et al,[40] 2010	Prospective Cohort	+	+	+	+	+	+	+	+	**8**
Vila-Canet et al,[41] 2021	Prospective Cohort	+	+	+	+	-	+	+	+	**7**
Rahman et al,[42] 2020	Prospective Cohort	+	+	+	+	-	+	+	+	**7**
Lafage et al,[43] 2021	Retrospective Cohort	-	+	+	+	++	+	+	+	**8**
Carreon et al,[44] 2020	Prospective Cohort	+	+	+	+	-	+	+	-	**6**
Tuomainen et al,[45] 2018	Prospective Cohort	+	+	+	+	++	+	+	-	**8**
Cushnie et al,[46] 2022	Prospective Cohort	+	+	+	+	++	+	+	-	**8**
Held et al,[47] 2022	Prospective Cohort	+	+	+	+	++	+	+	+	**9**
Sinikallio et al,[48] 2011	Prospective Cohort	-	+	+	+	++	+	+	+	**8**
Falavigna et al,[49] 2015	Prospective Cohort	-	+	+	+	-	+	+	+	**6**
Lee et al,[50] 2017	Retrospective Cohort	-	+	+	+	++	+	+	+	**8**
Maratos et al,[51] 2012	Prospective Cohort	+	+	+	+	-	+	+	+	**7**
Nayar et al,[52] 2017	Retrospective Cohort	-	+	+	+	++	+	+	+	**8**
Theologis et al,[53] 2016	Retrospective Cohort	-	+	+	+	++	+	+	+	**8**
Kobayashi et al,[54] 2019	Retrospective Cohort	-	+	+	+	-	+	+	+	**6**
Mayo et al,[55] 2017	Retrospective Cohort	-	+	+	+	++	+	+	+	**8**
Holbert et al,[56] 2022	Retrospective Cohort	-	+	+	+	++	+	+	-	**7**

© 2024 Javeed S et al. *JAMA Network Open*

(continued) **eTable 6: Study Quality and Risk of Bias using the Newcastle-Ottawa Scale**										
**Study Author, year**	**Study Design**	**Selection**				**Comparability**	**Outcome**			**Total Score**
		**Exposed Cohort Representative?**	**Selection of Non-Exposed Cohort**	**Ascertainment of Exposure**	**Outcome not present at study start?**	**Based on Design or Analysis**	**Assessment of Outcome**	**Timing of Follow-up (≥6 months)**	**Adequate Follow-up (≥80%)**	
Jacob et al,[57] 2022	Retrospective Cohort	-	+	+	+	-	+	+	-	**5**
Sangbong et al,[58] 2022	Retrospective Cohort	-	+	+	+	-	+	+	+	**6**
Patel et al,[59] 2022	Retrospective Cohort	-	+	+	+	+	+	+	-	**6**
Wang et al,[60] 2023	Retrospective Cohort	-	+	+	+	++	+	+	+	**8**

© 2024 Javeed S et al. *JAMA Network Open*

eTable 7: Inter-rater reliability of screened and extracted data		
**Variable**	**Cohen's Kappa[a] (95% CI)**	**Percentage of agreement between reviewers**
Inclusion decision	0.80 (0.67–0.92)	90%
Study design	1 (1–1)	100%
The number of study centers	1 (1–1)	100%
Level of evidence	0.65 (0.5–0.81)	82%
Surgery level	1 (1–1)	100%
Reported surgical details (yes/no)	0.95 (0.85–1)	97%
Reported missing data handling (yes/no)	1 (1–1)	100%
Multivariable analysis (yes/no)	1 (0.99–1)	100%
Adjusted age (yes/no)	1 (1–1)	100%
Adjusted sex (yes/no)	1 (0.99–1)	100%
Adjusted comorbidities (yes/no)	1 (1–1)	100%
Adjusted baseline symptom severity (yes/no)	0.95 (0.87–1)	97%
Adjusted sociodemographic variables (yes/no)	0.88 (0.72–1)	96%
Reported statistical methods (yes/no)	1 (1–1)	100%
Propensity score match (yes/no)	1 (1–1)	100%
Sensitivity analysis (yes/no)	1 (1–1)	100%
PROMs	NA	95%
Mental health methods	NA	100%

[a]Values ≤0 as indicating no agreement and 0.01–0.20 as none to slight, 0.21–0.40 as fair, 0.41– 0.60 as moderate, 0.61–0.80 as substantial, and 0.81–1.00 as almost perfect agreement.

© 2024 Javeed S et al. *JAMA Network Open*

eTable 8: Meta-Regressions to Identify the Variables Explaining I^2 in Improvement in PROMs in Patients with No-Depression *vs.* Depression

**Univariable Meta-Regressions**				
**Variable**	**β[a] (95% CI)**	***z***	**p value**	**R^2, %**
**Patient-level variables**				
Age at baseline, y	<0.01 (-0.01 to 0.01)	0.68	0.49	0
Sex, (male-female ratio)	<-0.01 (<-0.01 to <0.01)	-0.88	0.38	0
Percent of comorbidities, %	-0.46 (-0.71 to -0.20)	-3.53	<0.01	23
**Study-level variables**				
First year of patient enrollment	-0.01 (-0.03 to <0.01)	-1.89	0.06	7
Study quality (NOS[b] >6 points vs. <=6)	0.04 (-0.09 to 0.18)	0.63	0.53	0
Follow-up duration, months	<0.01 (<-0.01 to <0.01)	0.08	0.13	0
Follow-up attrition, %	<-0.01 (-0.01 to <0.01)	-1.88	0.05	10
Depression stratification method (diagnosis vs. scale)	-0.09 (-0.27 to 0.07)	-1.12	0.26	3
Number of factors adjusted (out of 5 factors total)[c]	<-0.01 (-0.04 to 0.03)	-0.03	0.98	0
**Intervention-level variables**				
Levels of surgery (multiple vs. single)	-0.11 (-0.25 to 0.03)	-1.59	0.11	6
Type of surgery (fusion+decompression vs. decompression only)	-0.01 (-0.15 to 0.12)	-0.22	0.82	0
Approach (anteroposterior vs. posterior only)	-0.01 (-0.16 to 0.13)	-0.21	0.83	0
**Multivariable Meta-Regression[d]**				
**Variable**	**β (95% CI)**	***z***	**p value**	**R^2, %**
Age at baseline, y	0.04 (0.02 to 0.05)	3.84	<0.01	75
Sex, (male-female ratio)	<0.01 (<0.01 to <0.01)	3.01	<0.01	
Percent of comorbidities, %	-0.60 (-1.17 to -0.04)	-2.09	0.03	
First year of patient enrollment	-0.07 (-0.11 to -0.02)	-3.09	<0.01	
Follow-up attrition, %	-0.01 (-0.02 to <-0.01)	-2.31	0.02	
Depression stratification method (diagnosis *vs.* scale)	0.09 (-0.23 to 0.41)	0.55	0.57	
Levels of surgery (multiple vs. single)	<-0.01 (-0.35 to 0.34)	-0.02	0.97	

[a] Positive beta (β) coefficient indicate larger and negative indicate smaller standardized mean difference between depressed and non-depressed patients.
[b] Newcastle Ottawa Scale (NOS) to measure the study quality; NOS calculated for all studies is given in eTable 6.
[c] Number of factors included were age, sex, comorbidities, baseline symptom severity, and sociodemographic variables adjusted in multivariable analysis as mentioned in Table 1.
[d] Only variables with $R^2>0$ were included in multivariable meta-regression analysis. Age and Sex (male-female ratio) were tested for their clinical importance and were attained in final model since they substantially improved variability explained (R^2 75% *vs.* 38%).

Abbreviations: β: beta coefficient; R^2: coefficient of determination; *z*: *z* value

© 2024 Javeed S et al. *JAMA Network Open*

eTable 9: Non-depression Mental Health Conditions Assessed in Included Studies		
**Domain**	**Measure**	**N (%)**
Anxiety	History/diagnosis of anxiety	4 (9)
	Hospital Anxiety and Depression Scale – Anxiety subscale	4 (9)
	Multidimensional Scale of Perceived Social Support – Somatic Anxiety	2 (4)
	Anxiety Sensitivity Index – 3	1 (2)
	Unspecified	1 (2)
Optimism	Revised Life Orientation Test	1 (2)
Catastrophizing	Coping Strategies Questionnaire's Catastrophizing Subscale	1 (2)
Kinesiophobia	Tampa Scale for Kinesiophobia	1 (2)
*Did not report enough data to be included in meta-regression or sensitivity analysis		

© 2024 Javeed S et al. *JAMA Network Open*

eReferences

1. Peters JL, Sutton AJ, Jones DR, Abrams KR, Rushton L. Contour-enhanced meta-analysis funnel plots help distinguish publication bias from other causes of asymmetry. *J Clin Epidemiol*. Oct 2008;61(10):991-6. doi:10.1016/j.jclinepi.2007.11.010
2. Peterson RA, Brown SP. On the use of beta coefficients in meta-analysis. *J Appl Psychol*. Jan 2005;90(1):175-81. doi:10.1037/0021-9010.90.1.175
3. Lewis-Beck M, Bryman A, Liao T. The SAGE Encyclopedia of Social Science Research Methods. SAGE Publications, Inc.; 2004. https://sk.sagepub.com/reference/socialscience
4. Lee H, Hübscher M, Moseley GL, et al. How does pain lead to disability? A systematic review and meta-analysis of mediation studies in people with back and neck pain. *Pain*. Jun 2015;156(6):988-997. doi:10.1097/j.pain.0000000000000146
5. Bowman NA. Effect Sizes and Statistical Methods for Meta-Analysis in Higher Education. *Research in Higher Education*. 2012/05/01 2012;53(3):375-382. doi:10.1007/s11162-011-9232-5
6. Silver NC, Dunlap WP. Averaging correlation coefficients: should Fisher's z transformation be used? *Journal of applied psychology*. 1987;72(1):146.
7. Chiarotto A, Maxwell LJ, Terwee CB, Wells GA, Tugwell P, Ostelo RW. Roland-Morris Disability Questionnaire and Oswestry Disability Index: Which Has Better Measurement Properties for Measuring Physical Functioning in Nonspecific Low Back Pain? Systematic Review and Meta-Analysis. *Phys Ther*. Oct 2016;96(10):1620-1637. doi:10.2522/ptj.20150420
8. Vianin M. Psychometric properties and clinical usefulness of the Oswestry Disability Index. *J Chiropr Med*. Dec 2008;7(4):161-3. doi:10.1016/j.jcm.2008.07.001
9. Chiarotto A, Maxwell LJ, Ostelo RW, Boers M, Tugwell P, Terwee CB. Measurement Properties of Visual Analogue Scale, Numeric Rating Scale, and Pain Severity Subscale of the Brief Pain Inventory in Patients With Low Back Pain: A Systematic Review. *J Pain*. Mar 2019;20(3):245-263. doi:10.1016/j.jpain.2018.07.009
10. Boonstra AM, Schiphorst Preuper HR, Reneman MF, Posthumus JB, Stewart RE. Reliability and validity of the visual analogue scale for disability in patients with chronic musculoskeletal pain. *Int J Rehabil Res*. Jun 2008;31(2):165-9. doi:10.1097/MRR.0b013e3282fc0f93
11. Chiarotto A, Terwee CB, Kamper SJ, Boers M, Ostelo RW. Evidence on the measurement properties of health-related quality of life instruments is largely missing in patients with low back pain: A systematic review. *J Clin Epidemiol*. Oct 2018;102:23-37. doi:10.1016/j.jclinepi.2018.05.006
12. Gornet MF, Copay AG, Sorensen KM, Schranck FW. Assessment of health-related quality of life in spine treatment: conversion from SF-36 to VR-12. *Spine J*. Jul 2018;18(7):1292-1297. doi:10.1016/j.spinee.2018.02.023
13. Fong DYT, Chan BKY, Li S, Wan CH, Kazis LE. Average and individual differences between the 12-item MOS Short-form Health Survey version 2 (SF-12 V.2) and the veterans RAND 12-item Health Survey (VR-12) in the Chinese population. *Health Qual Life Outcomes*. Jul 2 2022;20(1):102. doi:10.1186/s12955-022-02010-z
14. Wee CC, Davis RB, Hamel MB. Comparing the SF-12 and SF-36 health status questionnaires in patients with and without obesity. *Health Qual Life Outcomes*. Jan 30 2008;6:11. doi:10.1186/1477-7525-6-11
15. Kiely JM, Brasel KJ, Guse CE, Weigelt JA. Correlation of SF-12 and SF-36 in a trauma population. *J Surg Res*. May 15 2006;132(2):214-8. doi:10.1016/j.jss.2006.02.004
16. Shafshak TS, Elnemr R. The Visual Analogue Scale Versus Numerical Rating Scale in Measuring Pain Severity and Predicting Disability in Low Back Pain. *J Clin Rheumatol*. Oct 1 2021;27(7):282-285. doi:10.1097/rhu.0000000000001320
17. Zhou Y, Deng J, Yang M, et al. Does the Preoperative Depression Affect Clinical Outcomes in Adults With Following Lumbar Fusion?: A Retrospective Cohort Study. *Clin Spine Surg*. May 1 2021;34(4):E194-e199. doi:10.1097/bsd.0000000000001102

18. Toivonen L, Häkkinen A, Pekkanen L, Salonen A, Kautiainen H, Neva MH. Influence of Depressive Symptoms on the Outcome of Lumbar Spine Fusion-A 5-year Follow-up Study. *Spine (Phila Pa 1976)*. Mar 15 2021;46(6):408-412. doi:10.1097/brs.0000000000003803
19. Parrish JM, Jenkins NW, Massel DH, et al. The Perioperative Symptom Severity of Higher Patient Health Questionnaire-9 Scores Between Genders in Single-Level Lumbar Fusion. *Int J Spine Surg*. Feb 2021;15(1):62-73. doi:10.14444/8007
20. Goyal DKC, Stull JD, Divi SN, et al. Combined Depression and Anxiety Influence Patient-Reported Outcomes after Lumbar Fusion. *Int J Spine Surg*. Apr 2021;15(2):234-242. doi:10.14444/8008
21. Yoo JS, Hrynewycz NM, Brundage TS, et al. The Influence of Preoperative Mental Health on PROMIS Physical Function Outcomes Following Minimally Invasive Transforaminal Lumbar Interbody Fusion. *Spine (Phila Pa 1976)*. Feb 15 2020;45(4):E236-e243. doi:10.1097/brs.0000000000003236
22. Wagner A, Shiban Y, Wagner C, et al. Psychological predictors of quality of life and functional outcome in patients undergoing elective surgery for degenerative lumbar spine disease. *Eur Spine J*. Feb 2020;29(2):349-359. doi:10.1007/s00586-019-06106-x
23. Mayo BC, Narain AS, Hijji FY, Massel DH, Bohl DD, Singh K. Preoperative Mental Health May Not Be Predictive of Improvements in Patient-Reported Outcomes Following a Minimally Invasive Transforaminal Lumbar Interbody Fusion. *Int J Spine Surg*. Feb 2020;14(1):26-31. doi:10.14444/7003
24. Laratta J, Carreon LY, Buchholz AL, et al. Effects of preoperative obesity and psychiatric comorbidities on minimum clinically important differences for lumbar fusion in grade 1 degenerative spondylolisthesis: analysis from the prospective Quality Outcomes Database registry. *J Neurosurg Spine*. Jul 24 2020:1-8. doi:10.3171/2020.4.Spine20296
25. Jenkins NW, Parrish JM, Brundage TS, Hrynewycz NM, Yoo JS, Singh K. Validity of Patient Health Questionnaire-9 in Minimally Invasive Lumbar Interbody Fusion. *Spine (Phila Pa 1976)*. Jun 1 2020;45(11):E663-e669. doi:10.1097/brs.0000000000003361
26. Goh GS, Liow MHL, Yeo W, et al. Patients With Poor Baseline Mental Health May Experience Significant Improvements in Pain and Disability After Minimally Invasive Transforaminal Lumbar Interbody Fusion: A 5-Year Follow-up Study. *Clin Spine Surg*. Jun 2020;33(5):205-214. doi:10.1097/bsd.0000000000000912
27. Zakaria HM, Mansour TR, Telemi E, et al. Use of Patient Health Questionnaire-2 scoring to predict patient satisfaction and return to work up to 1 year after lumbar fusion: a 2-year analysis from the Michigan Spine Surgery Improvement Collaborative. *J Neurosurg Spine*. Aug 23 2019:1-8. doi:10.3171/2019.6.Spine1963
28. Patel DV, Yoo JS, Khechen B, et al. PHQ-9 Score Predicts Postoperative Outcomes Following Minimally Invasive Transforaminal Lumbar Interbody Fusion. *Clin Spine Surg*. Dec 2019;32(10):444-448. doi:10.1097/bsd.0000000000000818
29. Amaral V, Marchi L, Martim H, et al. Influence of psychosocial distress in the results of elective lumbar spine surgery. *J Spine Surg*. Sep 2017;3(3):371-378. doi:10.21037/jss.2017.08.05
30. Carreon LY, Djurasovic M, Dimar JR, 2nd, et al. Can the anxiety domain of EQ-5D and mental health items from SF-36 help predict outcomes after surgery for lumbar degenerative disorders? *J Neurosurg Spine*. Sep 2016;25(3):352-6. doi:10.3171/2016.2.Spine151472
31. Parker SL, Godil SS, Zuckerman SL, Mendenhall SK, Devin CJ, McGirt MJ. Extent of preoperative depression is associated with return to work after lumbar fusion for spondylolisthesis. *World Neurosurg*. Apr 2015;83(4):608-13. doi:10.1016/j.wneu.2014.12.018
32. Miller JA, Derakhshan A, Lubelski D, et al. The impact of preoperative depression on quality of life outcomes after lumbar surgery. *Spine J*. Jan 1 2015;15(1):58-64. doi:10.1016/j.spinee.2014.06.020
33. Adogwa O, Verla T, Thompson P, et al. Affective disorders influence clinical outcomes after revision lumbar surgery in elderly patients with symptomatic adjacent-segment disease, recurrent stenosis, or pseudarthrosis: clinical article. *J Neurosurg Spine*. Aug 2014;21(2):153-9. doi:10.3171/2014.4.Spine12668

© 2024 Javeed S et al. *JAMA Network Open*

34. Trief PM, Ploutz-Snyder R, Fredrickson BE. Emotional health predicts pain and function after fusion: a prospective multicenter study. *Spine (Phila Pa 1976)*. Apr 1 2006;31(7):823-30. doi:10.1097/01.brs.0000206362.03950.5b
35. Derby R, Lettice JJ, Kula TA, Lee SH, Seo KS, Kim BJ. Single-level lumbar fusion in chronic discogenic low-back pain: psychological and emotional status as a predictor of outcome measured using the 36-item Short Form. *J Neurosurg Spine*. Oct 2005;3(4):255-61. doi:10.3171/spi.2005.3.4.0255
36. Goh GS, Liow MHL, Yue WM, Tan SB, Chen JL. Are Patient-Reported Outcomes of Minimally Invasive Transforaminal Lumbar Interbody Fusion Influenced by Preoperative Mental Health? *Global Spine J*. May 2021;11(4):500-508. doi:10.1177/2192568220912712
37. Stull JD, Divi SN, Goyal DKC, et al. Preoperative Mental Health Component Scoring Is Related to Patient Reported Outcomes Following Lumbar Fusion. *Spine (Phila Pa 1976)*. Jun 15 2020;45(12):798-803. doi:10.1097/brs.0000000000003399
38. Pollock R, Lakkol S, Budithi C, Bhatia C, Krishna M. Effect of psychological status on outcome of posterior lumbar interbody fusion surgery. *Asian Spine J*. Sep 2012;6(3):178-82. doi:10.4184/asj.2012.6.3.178
39. Abbott AD, Tyni-Lenné R, Hedlund R. Leg pain and psychological variables predict outcome 2-3 years after lumbar fusion surgery. *Eur Spine J*. Oct 2011;20(10):1626-34. doi:10.1007/s00586-011-1709-6
40. Mokhtar SA, Sears WR. Preoperative Health-Related Quality of Life Scores as Predictor of Clinical Outcomes after Degenerative Lumbar Surgery. *The Spine Journal*. 2010;10(9):S97-S98. doi:10.1016/j.spinee.2010.07.259
41. Vilà-Canet G, Covaro A, Isart A, et al. Elective Lumbar Spine Surgery in Depressed Patients: Is it Worth it? *Int J Spine Surg*. Jun 2021;15(3):418-422. doi:10.14444/8062
42. Rahman R, Ibaseta A, Reidler JS, et al. Changes in patients' depression and anxiety associated with changes in patient-reported outcomes after spine surgery. *J Neurosurg Spine*. Jan 31 2020:1-20. doi:10.3171/2019.11.Spine19586
43. Lafage R, Ang B, Schwab F, et al. Depression Symptoms Are Associated with Poor Functional Status Among Operative Spinal Deformity Patients. *Spine (Phila Pa 1976)*. Apr 1 2021;46(7):447-456. doi:10.1097/brs.0000000000003886
44. Carreon LY, Jespersen AB, Støttrup CC, Hansen KH, Andersen MO. Is the Hospital Anxiety and Depression Scale Associated With Outcomes After Lumbar Spine Surgery? *Global Spine J*. May 2020;10(3):266-271. doi:10.1177/2192568219845662
45. Tuomainen I, Pakarinen M, Aalto T, et al. Depression is associated with the long-term outcome of lumbar spinal stenosis surgery: a 10-year follow-up study. *Spine J*. Mar 2018;18(3):458-463. doi:10.1016/j.spinee.2017.08.228
46. Cushnie D, Soroceanu A, Stratton A, et al. Outcome of spine surgery in patients with depressed mental states: a Canadian spine outcome research network study. *Spine J*. Oct 2022;22(10):1700-1707. doi:10.1016/j.spinee.2022.05.012
47. Held U, Burgstaller JM, Deforth M, Steurer J, Pichierri G, Wertli MM. Association between depression and anxiety on symptom and function after surgery for lumbar spinal stenosis. *Sci Rep*. Feb 18 2022;12(1):2821. doi:10.1038/s41598-022-06797-1
48. Sinikallio S, Aalto T, Airaksinen O, Lehto SM, Kröger H, Viinamäki H. Depression is associated with a poorer outcome of lumbar spinal stenosis surgery: a two-year prospective follow-up study. *Spine (Phila Pa 1976)*. Apr 15 2011;36(8):677-82. doi:10.1097/BRS.0b013e3181dcaf4a
49. Falavigna A, Righesso O, Teles AR, et al. Responsiveness of depression and its influence on surgical outcomes of lumbar degenerative diseases. *Eur J Orthop Surg Traumatol*. Jul 2015;25 Suppl 1:S35-41. doi:10.1007/s00590-015-1651-0
50. Lee J, Kim HS, Shim KD, Park YS. The Effect of Anxiety, Depression, and Optimism on Postoperative Satisfaction and Clinical Outcomes in Lumbar Spinal Stenosis and Degenerative Spondylolisthesis Patients: Cohort Study. *Clin Orthop Surg*. Jun 2017;9(2):177-183. doi:10.4055/cios.2017.9.2.177

© 2024 Javeed S et al. *JAMA Network Open*

51. Maratos EC, Trivedi R, Richards H, Seeley H, Laing RJ. Psychological distress does not compromise outcome in spinal surgery. *Br J Neurosurg*. Aug 2012;26(4):466-71. doi:10.3109/02688697.2011.644821
52. Nayar G, Elsamadicy AA, Zakare-Fagbamila R, Farquhar J, Gottfried ON. Impact of Affective Disorders on Recovery of Baseline Function in Patients Undergoing Spinal Surgery: A Single Institution Study of 275 Patients. *World Neurosurg*. Apr 2017;100:69-73. doi:10.1016/j.wneu.2016.12.098
53. Theologis AA, Ailon T, Scheer JK, et al. Impact of preoperative depression on 2-year clinical outcomes following adult spinal deformity surgery: the importance of risk stratification based on type of psychological distress. *J Neurosurg Spine*. Oct 2016;25(4):477-485. doi:10.3171/2016.2.Spine15980
54. Kobayashi Y, Ogura Y, Kitagawa T, et al. The influence of preoperative mental health on clinical outcomes after laminectomy in patients with lumbar spinal stenosis. *Clin Neurol Neurosurg*. Oct 2019;185:105481. doi:10.1016/j.clineuro.2019.105481
55. Mayo BC, Massel DH, Bohl DD, et al. Preoperative Mental Health is not Predictive of Patient-reported Outcomes Following a Minimally Invasive Lumbar Discectomy. *Clin Spine Surg*. Dec 2017;30(10):E1388-e1391. doi:10.1097/bsd.0000000000000466
56. Holbert SE, Wertz S, Turcotte J, Patton C. The Impact of Depression and Anxiety on Perioperative Outcomes and Patient-Reported Outcomes Measurement Information System Physical Function After Thoracolumbar Surgery. *Int J Spine Surg*. Dec 2022;16(6):1095-1102. doi:10.14444/8365
57. Jacob KC, Patel MR, Nie JW, et al. Presenting Mental Health Influences Postoperative Clinical Trajectory and Long-Term Patient Satisfaction After Lumbar Decompression. *World Neurosurg*. Aug 2022;164:e649-e661. doi:10.1016/j.wneu.2022.05.024
58. Ko S, Choi W. Usefulness of preoperative Short Form-36 Mental Component Score as a prognostic factor in patients who underwent decompression surgery for degenerative lumbar spinal stenosis. *Medicine (Baltimore)*. Sep 30 2022;101(39):e30231. doi:10.1097/md.0000000000030231
59. Patel MR, Jacob KC, Amin KS, et al. Does Baseline Mental Health Influence Outcomes among Workers' Compensation Claimants Undergoing Minimally Invasive Transforaminal Lumbar Interbody Fusion? *Asian Spine J*. Feb 2023;17(1):96-108. doi:10.31616/asj.2021.0388
60. Wang SK, Cui P, Wang DF, Wang P, Kong C, Lu SB. Preoperative Zung depression scale predicts outcomes in older patients undergoing short-segment fusion surgery for degenerative lumbar spinal disease. *Eur Spine J*. Feb 2023;32(2):718-726. doi:10.1007/s00586-022-07497-0

© 2024 Javeed S et al. *JAMA Network Open*
