# Supplementary material for: Implications of Preoperative Depression for Lumbar Spine Surgery Outcomes: A Systematic Review and Meta-Analysis
Source: JAMA Netw Open. 2024 Jan 26;7(1):e2348565. doi: 10.1001/jamanetworkopen.2023.48565 (PMC10818221; doi:10.1001/jamanetworkopen.2023.48565)
Supplement: Supplement 2. — Data Sharing Statement [file jamanetwopen-e2348565-s002.pdf]

Data Sharing Statement

Javeed. Implications of Preoperative Depression for Lumbar Spine Surgery Outcomes. *JAMA Netw Open*. Published January 26, 2024. doi:10.1001/jamanetworkopen.2023.48565

Data
Data available: Yes
Data types: Data (not involving human participants)
How to access data: The individual study data with a data dictionary defining each variable in the dataset can be made available after publication on a reasonable request for legitimate research purposes.
When available: With publication

Supporting Documents
Document types: Statistical/analytic code
How to access documents: Data Dictionary
When available: With publication

Additional Information
Who can access the data: Researchers with a legitimate research request
Types of analyses: Meta-analysis
Mechanisms of data availability: After a proposal
